# Dose-dependent reversal of KCC2 hypofunction and phenobarbital-resistant neonatal seizures by ANA12

**DOI:** 10.1038/s41598-018-30486-7

**Published:** 2018-08-10

**Authors:** B. M. Carter, B. J. Sullivan, J. R. Landers, S. D. Kadam

**Affiliations:** 1 Neuroscience Laboratory, Hugo Moser Research Institute at Kennedy Krieger, Baltimore, MD USA; 2 Department of Neurology, Johns Hopkins University School of Medicine, Baltimore, MD 21205 USA

## Abstract

Neonatal seizures have an incidence of 3.5 per 1000 newborns; while hypoxic-ischemic encephalopathy (HIE) accounts for 50–60% of cases, half are resistant to 1st-line anti-seizure drugs such as phenobarbital (PB). Tyrosine receptor kinase B (TrkB) activation following ischemic injury is known to increase neuronal excitability by downregulation of K-Cl co-transporter 2 (KCC2); a neuronal chloride (Cl^−^) co-transporter. In this study, three graded doses of ANA12, a small-molecule selective TrkB antagonist, were tested in CD1 mice at P7 and P10 following induction of neonatal ischemia by a unilateral carotid ligation. The PB loading dose remained the same in all treatment groups at both ages. Evaluation criteria for the anti-seizure efficacy of ANA12 were: (1) quantitative electroencephalographic (EEG) seizure burden and power, (2) rescue of post-ischemic KCC2 and pKCC2-S940 downregulation and (3) reversal of TrkB pathway activation following ischemia. ANA12 significantly rescued PB resistant seizures in a dose-dependent manner at P7 and improved PB efficacy at P10. Additionally, female pups responded better to lower doses of ANA12 compared to males. ANA12 significantly reversed post-ischemic KCC2 downregulation and TrkB pathway activation at P7 when PB alone was inefficacious. Rescuing KCC2 hypofunction may be critical for preventing emergence of refractory seizures.

## Introduction

Phenobarbital (PB), a barbiturate that prolongs the opening of GABA receptors, remains the most common first-line anti-seizure drug (ASD). Ischemic neonatal seizures are often refractory to PB and unresponsive to adjunct ASDs^1^. There is no consensus on evidence based treatment protocols when PB fails^2–5^. Hypoxic-ischemic encephalopathy (HIE) remains a major cause of neonatal seizures, more than half present electrographic seizures even after treatment with PB and hypothermia^6–8^. Depolarizing GABAergic signaling in the immature brain has been proposed to underlie PB refractoriness^9–12^.

The neuronal Cl^−^ gradient dictates GABA-dependent neuronal hyperpolarization and is maintained by members of the SLC12A family of cation-chloride co-transporters^13^. Potassium-chloride cotransporter 2 (KCC2) is the primary neuronal Cl^−^ extruder and thus maintains the [Cl^−^]_i_ gradient that allows strong, hyperpolarizing GABAergic inhibition^14^. KCC2 hypofunction results in decreased inhibition and increased network hyperexcitability underlying numerous disease states including epilepsy, neuropathic pain, neuropsychiatric disorders, and autism^15–20^. KCC2 loss of function mutations underlies infantile-onset pharmacoresistant epilepsy^21,22^. Additionally, phosphorylation of KCC2 at serine 940 (pKCC2-S940) has been shown to modulate membrane localization and thus function^23,24^.

Acute KCC2 downregulation is one of the hallmarks of excitotoxic neuronal injuries like neonatal ischemic insults and may underlie the emergence of PB refractoriness^25,26^. Rapid increases in the TrkB ligand BDNF have been reported following ischemia^27,28^. Activation of TrkB by phosphorylation at tyrosine-816 (pTrkB-T816) has been shown to induce phospholipase C gamma1 (PLCγ1) phosphorylation (pPLCγ1-T783); this has been linked to KCC2 downregulation and epileptogenesis^29–32^.

Vesicular glutamate transporters (vGLUTs) are responsible for loading glutamate into synaptic vesicles. The loss of vGLUT reduces glutamatergic neurotransmission, causing severe deficits to cognition, and increases in seizure susceptibility^33,34^. Previous studies have demonstrated the bilateral increase in vGLUT1 and unaltered levels of vesicular GABA transporter (vGAT) following unilateral ischemia in Sprague-Dawley rats^35^. To investigate vGLUT, and contrast it with vGAT, both were quantified in this neonatal ischemic seizure model.

ANA12, a small-molecule TrkB antagonist^36^ crosses the blood brain barrier efficiently. BDNF binding to TrkB following energy deprivation, and the subsequent KCC2 downregulation has demonstrated a causal relationship^26^. This study examined ANA12’s dose-dependent efficacy to rescue PB resistant seizures, in both male and female CD-1 pups at two distinct neonatal ages at which PB switches from being inefficacious to efficacious. A single dose of ANA12 + PB was given at P7 when seizures are PB-nonresponsive and at P10 when seizures are PB-responsive. This dichotomy of PB-responses in the CD-1 model likely represents the developmental switch in GABA acting initially as a depolarizing agent vs. as a hyperpolarizing agent in more mature neurons. Deviation from this natural progression, due to KCC2 hypofunction induced by HIE, may underlie the transient appearance of pharmacoresistant seizures that regress over a period of a few days^37^.

This study investigated the efficacy of graded doses of ANA12 (Table 1) in modulating the TrkB pathway and associated KCC2 hypofunction, in a model of neonatal ischemic seizures.

**Table 1:** Sample Sizes for P7 and P10.

P7	ANA 0.5 mg/kg	ANA 2.5 mg/kg	ANA 5 mg/kg	PB alone
Male	9	13	13	13
Female	11	11	10	11
Total	20	24	23	24
**P10**	**ANA 0.5 mg/kg**	**ANA 2.5 mg/kg**	**ANA 5 mg/kg**	**PB alone**
Male	6	7	6	6
Female	5	5	5	5
Total	11	12	11	11

## Results

### ANA12 rescues PB-resistant seizures in a dose-dependent manner at P7

**Figure 1:**
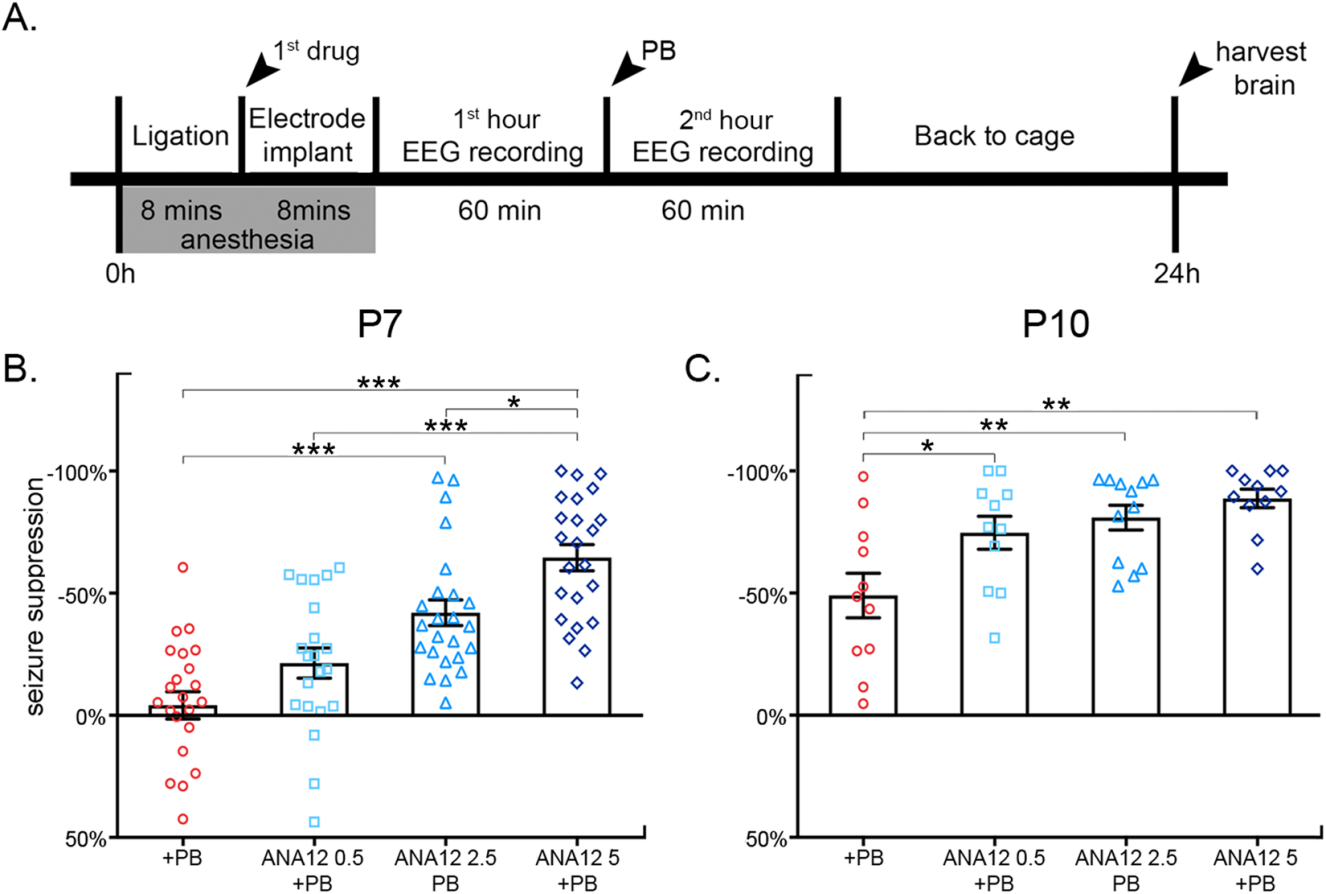
(**A**) Experimental paradigm (**B**) PB inefficacy at P7, previously characterized in this ischemic model of neonatal seizures, was replicated in this study with a mean seizure suppression of ~4%. An acute single IP administration of ANA12 administered immediately post-ligation rescued PB-refractoriness in a dose-dependent manner. PB-efficacy improved by 22% with 0.5 mg/kg ANA12, 42% with 2.5 mg/kg ANA12, and 65% with 5 mg/kg ANA12. Mean seizure suppression resulting from 5 mg/kg of ANA12 was significantly greater than that achieved by both 0.5 mg/kg and 2.5 mg/kg of ANA12. (**C**) P10 seizures were PB-responsive. All three doses of ANA12 significantly improved mean seizure suppression compared to PB alone at P10. Between-group comparison was done using one-way ANOVAs; *P < 0.05, **P < 0.01, ***P < 0.001.

The dose-dependent effects of three graded doses of ANA12 + PB were evaluated at P7. In the absence of ANA12, PB failed as an anti-seizure agent, replicating previous results for the CD-1 mouse model^38^. 0.5 mg/kg of ANA12 + PB suppressed seizures by −21.8 ± 6.5%, 2.5 mg/kg by −42 ± 5.3%, and 5 mg/kg by −64.6 ± 5.4% (*F*_3,87_ = 22.087 P ≤ 0.001, one-way ANOVA; post-hoc, ANA12 0.5 mg/kg: p = 0.205, ANA12 2.5 mg/kg: p ≤ 0.001; ANA12 5 mg/kg: p ≤ 0.001; vs. PB; Fig. 1B, Figure supplementary video 1). 5 mg/kg of ANA12 + PB significantly improved seizure suppression compared to 0.5 mg/kg and 2.5 mg/kg doses of ANA12 + PB (*F*_3,87_ = 22.087 P ≤ 0.001, one-way ANOVA; post-hoc, ANA12 0.5 mg/kg: p ≤ 0.001, ANA 2.5 mg/kg: p = 0.028; Fig. 1B).

**Figure 2:**
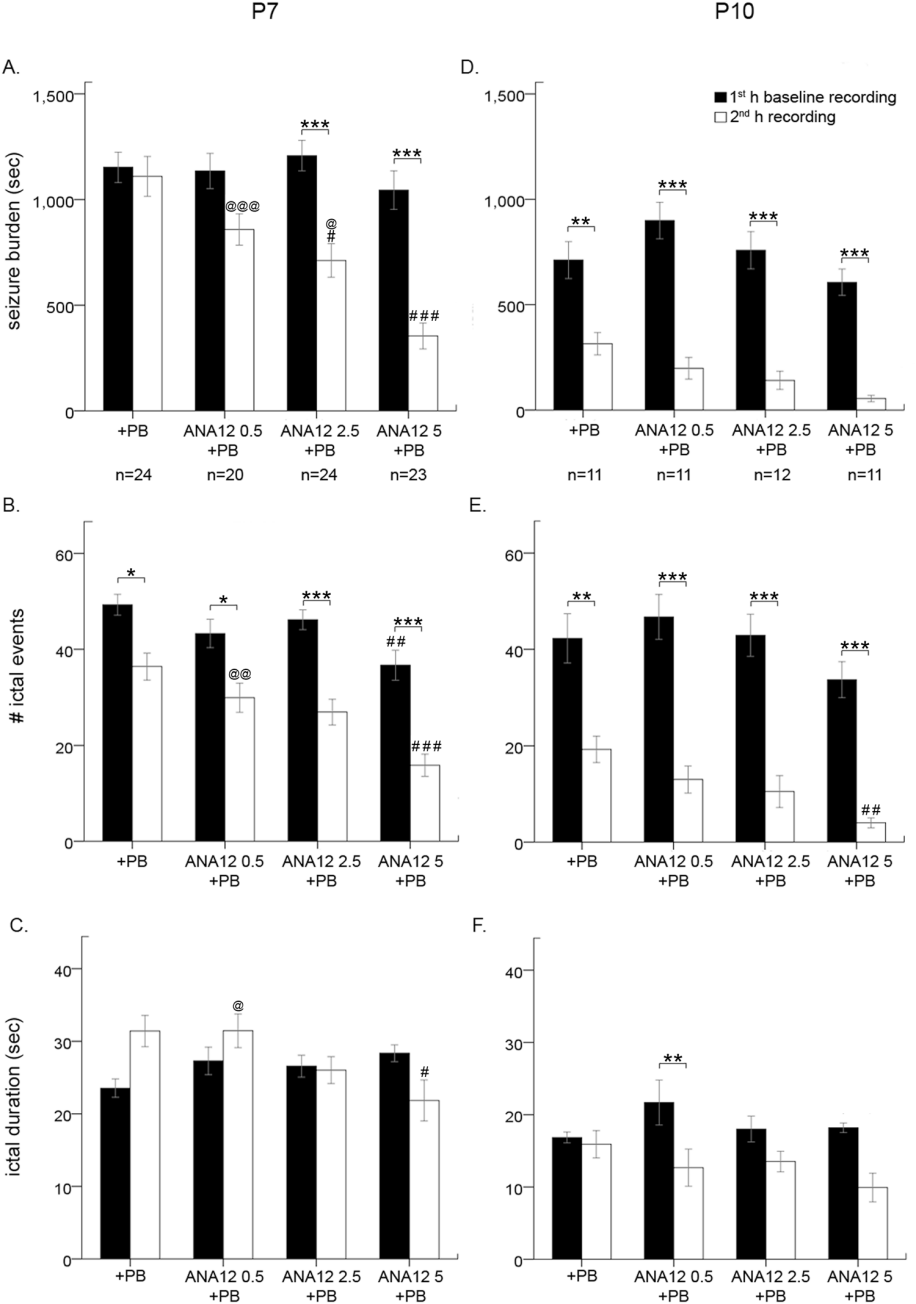
Baseline and post-PB seizure burden, ictal events and durations at P7 and P10 (**A**) P7 seizures were PB-resistant. ANA12 given IP immediately after the carotid ligation reversed PB-resistance in a dose-dependent manner at P7. (**B**) At P7, number of ictal events were reduced significantly with PB and further with each graded dose of ANA12 + PB. (**C**) Ictal durations were not significantly modulated by any treatment. (**D**) At P10, seizures were PB-responsive. ANA12 improved PB-efficacy with each graded dose. (**E**) At P10, number of ictal events were reduced significantly with PB alone, and further reduced per graded increase in dose of ANA12 + PB at P10 that was significant only at 5 mg/kg dose. (**F**) Only 0.5 mg/kg on ANA12 significantly decreased ictal duration at P10. All comparisons done via two-way ANOVA; bars with “*” denote significance between 1^st^ and 2^nd^ hour within a treatment (*P < 0.05, **P < 0.01, ***P < 0.001). Bars with “#” denote significance between groups compared to PB (^#^*P* < 0.05, ^##^*P* < 0.01, ^###^*P* < 0.001). Bars with “@” denote significance between groups compared to ANA 5 + PB (^@^*P* < 0.05, ^@@^*P* < 0.01, ^@@@^*P* < 0.001).

PB alone resulted in an average seizure burden of 1152.5 ± 71.9 seconds in the first hour and 1109.6 ± 94.4 seconds in the second (post-PB) hour (Fig. 2A). ANA12 increased PB efficacy in a dose-dependent manner; both 2.5 mg/kg and 5 mg/kg significantly reduced second hour seizure burden (for stats, see Table 2A). Ictal events, but not ictal durations, were significantly decreased by PB alone and ANA12 + PB in a dose-dependent manner (Fig. 2B,C; for detailed stats for each treatment group, see Table 2A).

**Table 2:** EEG Seizure Statistics (*P < 0.05, **P < 0.01, ***P < 0.001).

A) P7 seizure stats	+PB	0.5 mg/kg ANA12 + PB	2.5 mg/kg ANA12 + PB	5 mg/kg ANA12 + PB
1st hr seizure burden	1152.5 ± 71.9	1135.0 ± 83.5	1207.5 ± 71.9	1044.4 ± 90.9
2nd hr seizure burden	1109.6 ± 94.4	858.5 ± 74.3	711.7 ± 79.7	354.4 ± 61.3
2 way ANOVA F_3, 174_ = 6.145; P = 0.0005*** post-hoc 1st vs 2nd hr	p > 0.9999	p = 0.62	p = 0.0003***	p ≤ 0.0001***
post-hoc vs 5 mg/kg	p < 0.0001***	p = 0.0006***	p = 0.041*	N/A
1st hr ictal events	49.3 ± 2.2	43.3 ± 3.0	46.2 ± 2.1	36.7 ± 3.1
2nd hr ictal events	36.42 ± 2.8	30.0 ± 3.0	27.0 ± 2.7	15.9 ± 2.3
2 way ANOVA: F_3,174_ = 1.169; P = 0.3229 post-hoc 1st hr vs 2nd hr	p = 0.0143*	p = 0.0275*	p ≤ 0.0001***	p ≤ 0.0001***
1st hr ictal duration	23.54 ± 1.27	27.31 ± 1.90	26.57 ± 1.51	28.35 ± 1.16
2nd hr ictal duration	31.41 ± 2.16	31.45 ± 2.31	26.03 ± 1.86	21.84 ± 2.81
2 way ANOVA: F_3,174_ = 5.161; P = 0.0019** post-hoc 1st hr vs 2nd hr	p = 0.0964	p > 0.9999	p > 0.9999	p = 0.4857
**B) P10 seizure stats**	**+PB**	**0.5 mg/kg ANA12 + PB**	**2.5 mg/kg ANA12 + PB**	**5 mg/kg ANA12 + PB**
1st hr seizure burden	711.8 ± 87.4	899.1 ± 86.4	758.3 ± 88.6	606.4 ± 62.7
2nd hr seizure burden	314.6 ± 53.1	198.2 ± 50.9	140.8 ± 43.2	54.6 ± 15.3
2 way ANOVA F_3,82_ = 1.903; P = 0.1356 post-hoc 1st vs 2nd hr	p = 0.0018**	p ≤ 0.0001***	p ≤ 0.0001***	p ≤ 0.0001***
1st hr ictal events	42.3 ± 5.1	46.7 ± 4.6	42.9 ± 4.4	33.7 ± 3.7
2nd hr ictal events	19.3 ± 2.7	13 ± 2.8	10.5 ± 3.3	4.0 ± 1.0
2 way ANOVA: F_3,174_ = 1.169; P = 0.3229 post-hoc 1st hr vs 2nd hr	p = 0.0011**	p ≤ 0.0001***	p ≤ 0.0001***	p ≤ 0.0001***
1st hr ictal duration	16.85 ± 0.75	21.69 ± 3.11	18.02 ± 1.79	18.20 ± 0.65
2nd hr ictal duration	15.91 ± 1.88	12.68 ± 2.58	13.53 ± 1.14	9.93 ± 1.99
2 way ANOVA: F_3,174_ = 5.161 P = 0.0019** post-hoc 1st hr vs 2nd hr	p > 0.9999	p = 0.0375*	p > 0.9999	p = 0.0867

### ANA12 significantly improves PB-efficacy at P10

ANA12 was evaluated as an adjunct to PB at P10, when PB is efficacious (Fig. 2D)^38^. PB alone suppressed seizures by −49 ± 9.1%; 0.5 mg/kg of ANA12 + PB significantly improved seizure suppression by −74.7 ± 6.7%, 2.5 mg/kg by −80.9 ± 5.1%, and 5 mg/kg by −88.8 ± 3.8% (*F*_3,41_ = 7.042 P = 0.001, one-way ANOVA; post-hoc, ANA12 0.5 mg/kg: p = 0.047; ANA12 2.5 mg/kg: p = 0.006; ANA12 5 mg/kg: p = 0.001; vs. PB; Fig. 1C). Within groups, PB alone and all three doses of ANA12 + PB significantly suppressed seizures in the 2^nd^ h. At P10, PB alone was efficacious as an ASD; ANA12 worked as an effective adjunct drug to improve seizure suppression by PB. Similar to findings at P7, both PB alone and all doses of ANA12 + PB decreased ictal events but not ictal durations at P10 (Fig. 2E,F; for stats, see Table 2B).

### EEG power was not a reliable indicator of dose-dependent seizure suppression

**Figure 3:**
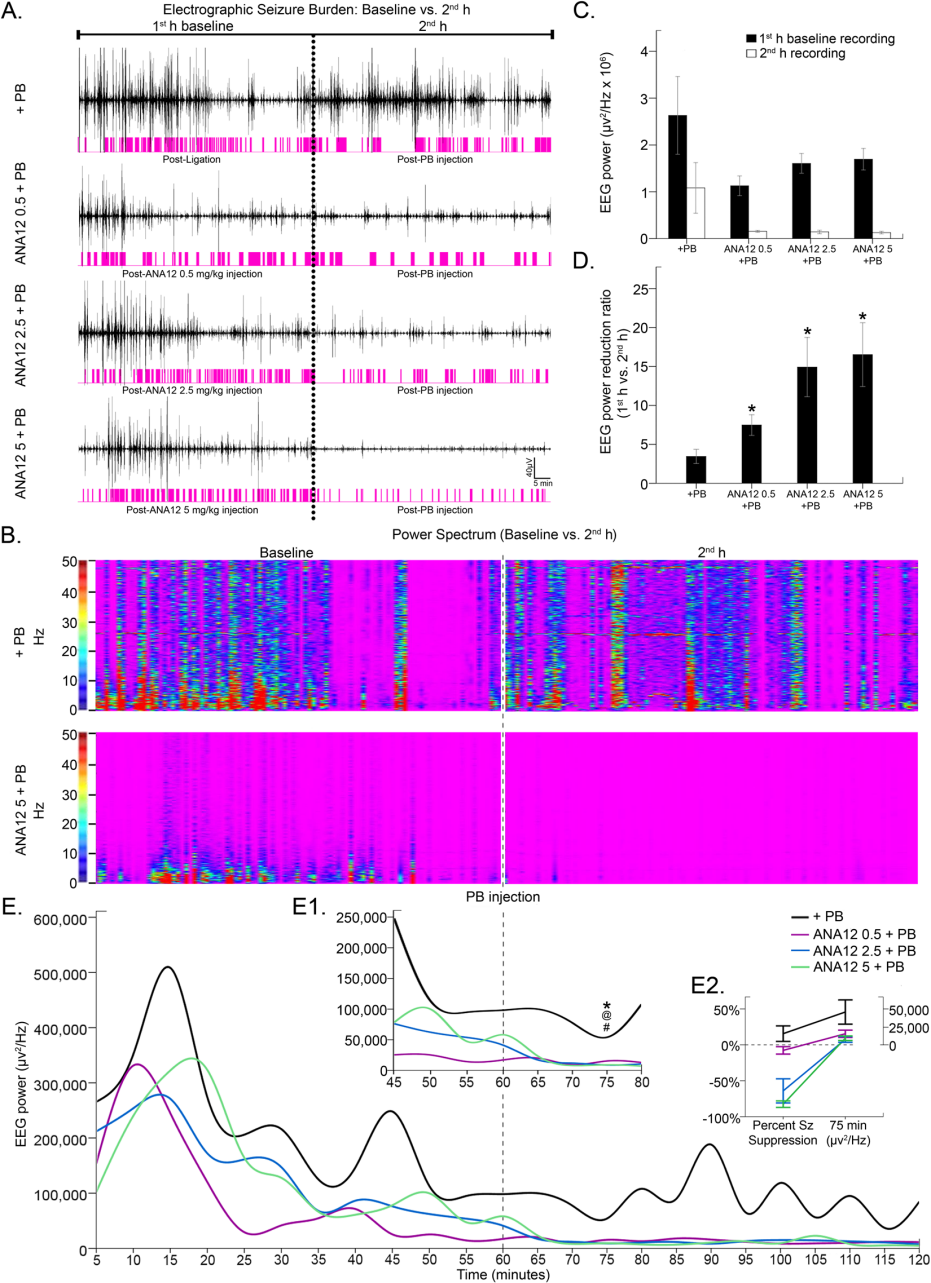
P7 EEG power analysis. (**A**) ANA12 + PB reduced electrographic seziure burden in a dose-dependent manner. (**B**) Power spectrum of PB alone and 5 mg/kg of ANA12 + PB. (**C**) No groups had significantly reduced EEG power in the 2^nd^ h. All comparisions done via two-way ANOVA. (**D**) All doses of ANA12 + PB significantly increased the EEG power reduction ratio compared to PB alone. (Between group comparisons to PB alone were done using independent *t*-tests; *P < 0.05, **P < 0.01, ***P < 0.001.) (**E**) Mean EEG power over the course of the 2 h recordings. (E1) 15 minutes post-PB injection showed ANA12 + PB significantly reduced EEG power at all doses. (Between group comparisons to PB alone were done with one-way ANOVAs; ANA12, 0.5 mg/kg: *P < 0.05; ANA12, 2.5 mg/kg: ^@^*P* < 0.01; ANA12 5 mg/kg: ^#^*P* < 0.05). (E2) Line graphs show percent seizure suppression and associated EEG powers for each treatment group at 15 minutes post-PB injection. All doses of ANA12 + PB had significant associations between seizure suppression and EEG power reduction at 15 min post-PB injection and PB alone did not. Percent seizure suppression vs. EEG power was done using paired *t*-tests; ^γ^*P* < 0.05, ^γγ^*P* < 0.01, ^γγγ^*P* < 0.001.

Previous studies have used EEG power to ascertain anti-seizure efficacy of proposed AEDs^9,39^. In this study, EEG power failed to accurately mimic actual EEG seizure burden data (Fig. 3A,C, Figure supplement 1A–D). There were no significant differences in 1^st^ versus 2^nd^ h power within any treatment groups [1^st^ h vs 2^nd^ h: F_3,40_ = 0.3151 P = 0.8144, two-way ANOVA, post hoc: PB alone: 2631972.9 ± 831527.4 µv^2^/Hz vs. 1082182.9 ± 539356.0 µv^2^/Hz (p = 0.1602); ANA12 0.5 mg/kg: 1129408.7 ± 210547.1 vs. 154533.5 ± 20314.1 µv^2^/Hz (p > 0.9999), ANA12 2.5 mg/kg: 1606571.4 ± 211424.7 vs. 142410 ± 38506.5 µv^2^/Hz (p = 0.1295), ANA12 5 mg/kg: 1694877.5 ± 229543.3 µv^2^/Hz vs. 128060.9 ± 27283.2 µv^2^/Hz (p = 0.1472); Fig. 3B,C)]. EEG power was a poor measure of seizure suppression in general and had no resolution to detect dose-dependent efficacies.

Power reduction ratios were calculated to normalize each animal’s 2^nd^ hour power to its own 1^st^ hour baseline (Fig. 3D). All doses of ANA12 + PB significantly reduced EEG power ratios (1^st^ h to 2^nd^ h) compared to PB alone [ANA12 0.5 mg/kg: 7.5 ± 1.3 (*t*_10_ = −2.498 p = 0.032), ANA12 2.5 mg/kg: 14.9 ± 3.8 (*t*_5.578_ = −2.932 p = 0.029), ANA12 5 mg/kg: 16.5 ± 4.1 (*t*_10_ = −3.114 p = 0.011); vs PB; independent *t*-test; Fig. 3D]. EEG power ratio data suggested that all doses of ANA12 were equally effective; however, 0.5 mg/kg had no significant effect on seizure suppression and 5 mg/kg was significantly better than 2.5 mg/kg (Fig. 1B). Therefore, EEG power alone could not detect the dose-dependent efficacy of ANA12 + PB.

Repeated measures ANOVA showed that all doses of ANA12 + PB significantly reduced EEG power (Fig. 3E1) at the epoch representing the 15 minute time point after PB injection (*F*_3,20_ = 6.176 P = 0.004, one-way ANOVA; post-hoc, ANA12 0.5 mg/kg: p = 0.031; ANA12 2.5 mg/kg: p = 0.009; ANA12 5 mg/kg: p = 0.010; vs. PB). All other epochs remained non-significant. Percent seizure suppression based on EEG seizure burdens and associated EEG power reduction at this time point (percent seizure suppression vs. EEG power at 75 min within treatment groups) was only significant between ANA12 0.5 mg/kg and 2.5 mg/kg (F_3,40_ = 22.83 P < 0.0001, two-way ANOVA; post-hoc, p = 0.0024; Fig. 3E2) indicating erroneous interpretation if EEG power was solely relied upon. Therefore, EEG power failed to demonstrate the differences in seizure burden between doses of ANA12 + PB (Figure supplement 1A–D), and thus failed as an indicator of ANA12’s dose-dependent seizure suppression. EEG power alone should not be used to gauge anti-seizure efficacies between animals.

### ANA12 rescued P7 post-ischemic KCC2 downregulation in a dose-dependent manner

**Figure 4:**
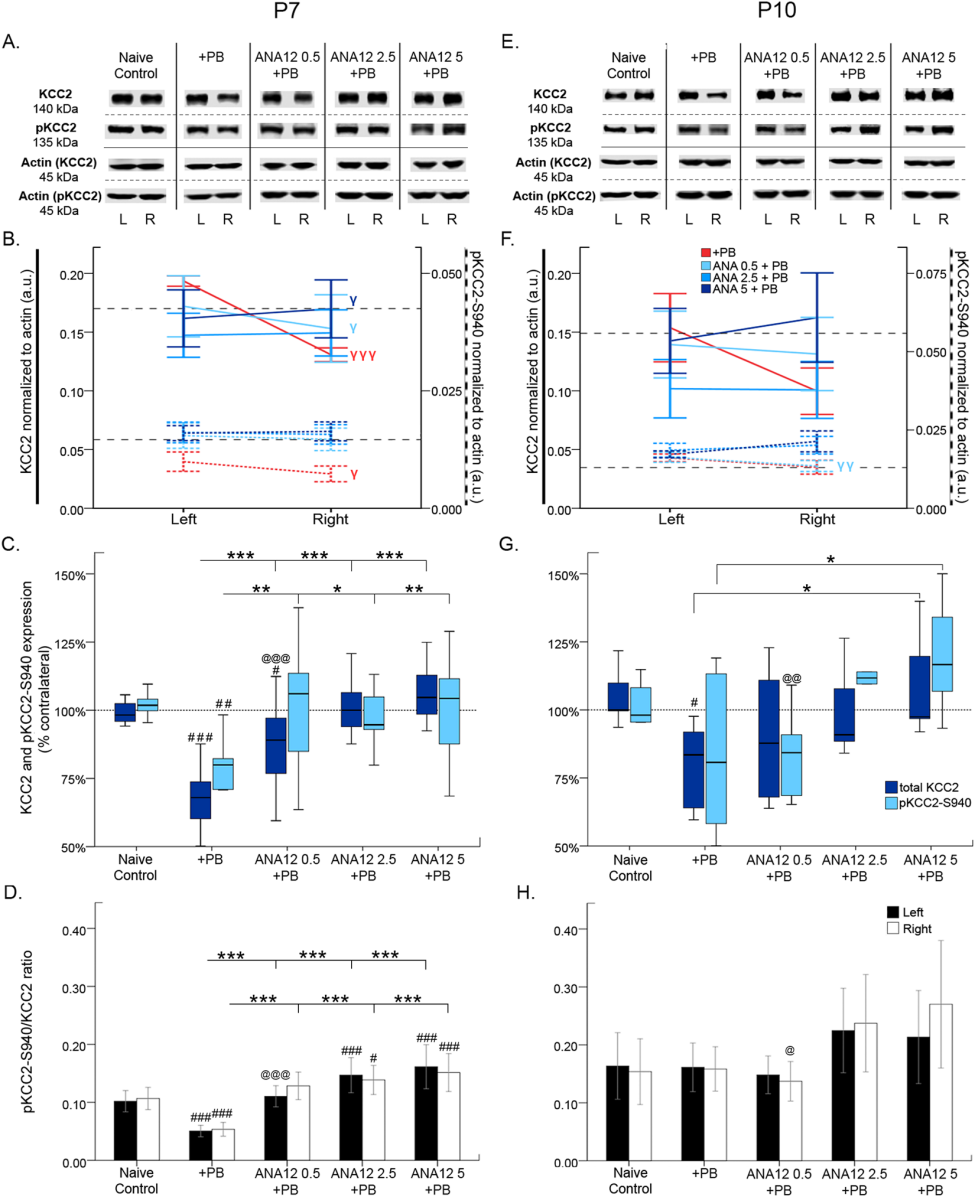
KCC2 and pKCC2-S940 expression 24 h post-ischemia. (**A** & **E**) Representative KCC2 and pKCC2 expression at P7 and P10. The blots presented for each group were cropped from separate gels for improvement in clarity and conciseness of presentation. Vertical solid black lines delineate the separate blots between groups, while horizontal dotted black lines delineate separate proteins run on different gels for the same sample. All gels were run in the same experimental conditions (see methods for details). (Full-length blots presented in Supplementary Figure 4). (**B** & **F**) Line graphs represent KCC2 and pKCC2 expression in the left and right hemispheres at P7 and P10. (**C** & **G**) Boxplots represent mean expression of KCC2 and pKCC2 in ipsilateral hemisphere normalized to the contralateral at 24 h after ischemia at P7 and P10. P7 mice from the PB alone group showed ~30% downregulation of KCC2 expression similar to previous reports. The post-ischemic downregulation of KCC2 expression was rescued by of ANA12 + PB tested in a dose-dependent manner in this study. The rescue of post-ischemic downregulation of KCC2 was associated with a significant upregulation of pKCC2 at all three ANA12 + PB doses tested. Within-group comparison of ipsilateral and contralateral hemispheres was done using pair-wise t-tests; γP < 0.05, γγP < 0.01, γγγP < 0.001. Between-group comparison to PB alone was done using one-way ANOVA for P7, and independent *t*-tests for P10; *P < 0.05, **P < 0.01, ***P < 0.001. Bars with “#” denote significance between group comparisons to naïve control using one-way ANOVA for P7 and independent *t*-tests for P10; ^#^P < 0.05, ^##^P < 0.01, ^###^P < 0.001. Bars with “@” denote significance between groups compared to ANA12 5 + PB using one-way ANOVA for P7 and independent *t*-tests for P10; ^@^P < 0.05, ^@@^P < 0.01, ^@@@^P < 0.001. (**D** & **H**) Bar graphs represent the ratio of pKCC2 to total KCC2 expression in the left and right hemispheres at 24 h post-ischemia P7 and P10. The ratio of normalized pKCC2 to normalized KCC2 was calculated for the same pup. (Between-group comparisons were done using one-way ANOVA; Post-hoc Bonferroni; *P < 0.05, **P < 0.01, ***P < 0.001 compared to PB-alone. Bars with “#” denote significance between group comparisons to Naïve Control; ^#^P < 0.05, ^##^P < 0.01, ^###^P < 0.001). For 1 h KCC2/pKCC2 WB data see Suppl. Figure 2.

24 h post-P7 ischemia, the effect of TrkB inhibition on KCC2 was evaluated (Fig. 4). Within groups, PB alone and 0.5 mg/kg of ANA12 + PB each had significantly less KCC2 in the right ischemic hemisphere compared to the left hemisphere (PB alone: *t*_11_ = 9.106 p ≤ 0.001; ANA12 0.5 mg/kg: *t*_15_ = 2.314 p = 0.035; paired *t*-test; Fig. 4A and B). Only 5 mg/kg of ANA12 + PB significantly rescued KCC2 expression in the right hemisphere compared to the left hemisphere (ANA12 5 mg/kg: *t*_15_ = −2.316 p = 0.035; paired *t*-test; Fig. 4A,B).

PB alone only had 67.9 ± 3.3% contralateral KCC2 expression, a ~30% decrease that is similar to previous reports (Kang *et al*., 2015a; Fig. 4A and C). Between groups, PB alone had significantly less percent contralateral KCC2 expression than naive controls (*F*_4,67_ = 27.160 P ≤ 0.001, one-way ANOVA; post-hoc, p ≤ 0.001; Fig. 4A and C). ANA12 + PB significantly rescued percent contralateral KCC2 expression at all doses tested (*F*_4,67_ = 27.160 P ≤ 0.001, one-way ANOVA; post-hoc, ANA12 0.5 mg/kg: 87.7 ± 3.6% (p ≤ 0.001); ANA12 2.5 mg/kg: 101 ± 2.8% (p ≤ 0.001); ANA12 5 mg/kg: 106.4 ± 2.3% (p ≤ 0.001); compared to PB alone; Fig. 4A and C). 2.5 mg/kg and 5 mg/kg of ANA12 + PB had significantly greater percent contralateral KCC2 expression than 0.5 mg/kg of ANA12 + PB (*F*_4,67_ = 27.160 P ≤ 0.001, one-way ANOVA; post-hoc, ANA 2.5 mg/kg: p = 0.011; ANA12 5 mg/kg: p ≤ 0.001; vs ANA12 0.5 mg/kg; Fig. 4A and C). 24 h post-P7 ischemia, ANA12 + PB efficaciously rescued downregulation of KCC2 in a dose-dependent manner.

24 h post-P7 ischemia, the effect of TrkB inhibition on post-ischemic pKCC2 was evaluated (Fig. 4A–C). Within groups, PB alone group showed significant dephosphorylation of pKCC2 in the right hemisphere compared to the left (*t*_8_ = 2.930 p = 0.019, paired *t*-test; Fig. 4A,B). There were no significant differences between right and left pKCC2 within all three ANA12 + PB groups. Between groups, PB alone had significantly less percent contralateral pKCC2 expression compared to naïve controls [% contralateral pKCC2: 75.6 ± 5.0% (*F*_4,55_ = 4.875 P = 0.002, one-way ANOVA; post-hoc, p = 0.002); Fig. 4C]. All three doses of ANA12 + PB significantly rescued ischemic downregulation of percent contralateral pKCC2 expression [*F*_4,55_ = 4.875 P = 0.002, one-way ANOVA; post-hoc, ANA12 0.5 mg/kg: 100.9 ± 6.0% (p = 0.004); ANA12 2.5 mg/kg: 97.4 ± 2.8% (p = 0.035); ANA12 5 mg/kg: 100.8 ± 5.6% (p = 0.009); vs. PB; Fig. 4A and C].

All three doses of ANA12 significantly rescued pKCC2/KCC2 ratios in both hemispheres (Fig. 4D; for stats, see Table 3A).

**Table 3:** Western Blot Statistics (*P < 0.05, **P < 0.01, ***P < 0.001).

A) P7 pKCC2/KCC2 ratio	Naïve	+PB	0.5 mg/kg ANA12 + PB	2.5 mg/kg ANA12 + PB	5 mg/kg ANA12 + PB
L pKCC2/KCC2 ratio	0.102 ± 0.0184	0.051 ± 0.0098	0.110 ± 0.0182	0.147 ± 0.0302	0.162 ± 0.0380
R pKCC2/KCC2 ratio	0.107 ± 0.0191	0.054 ± 0.0118	0.092 ± 0.236	0.139 ± 0.0251	0.108 ± 0.326
L pKCC2/KCC2 ratio 1 way ANOVA F_4,55_ = 30.88; P < 0.0001*** post-hoc vs PB alone	p < 0.0001***	N/A	p < 0.0001***	p < 0.0001***	p < 0.0001***
R pKCC2/KCC2 ratio 1 way ANOVA F_4,55_ = 25.96; P < 0.0001*** post-hoc vs PB alone	p < 0.0001***	N/A	p < 0.0001***	p < 0.0001***	p < 0.0001***
**B) P10 pKCC2/KCC2 ratio**	**Naïve**	**+PB**	**0.5 mg/kg ANA12 + PB**	**2.5 mg/kg ANA12 + PB**	**5 mg/kg ANA12 + PB**
L pKCC2/KCC2 ratio	0.164 ± 0.0574	0.161 ± 0.0420	0.148 ± 0.0326	0.225 ± 0.0729	0.214 ± 0.0802
R pKCC2/KCC2 ratio	0.154 ± 0.0568	0.158 ± 0.0382	0.137 ± 0.0341	0.237 ± 0.0834	0.270 ± 0.1100
L pKCC2/KCC2 ratio 1 way ANOVA: F_4,26_ = 2.312; P = 0.0844 post-hoc vs PB Alone	p > 0.9999	N/A	p > 0.9999	p = 0.3432	p = 0.7202
R pKCC2/KCC2 ratio 1 way ANOVA: F_4,25_ = 4.103; P = 0.0108* post-hoc vs PB Alone	p > 0.9999	N/A	p > 0.9999	p = 0.4362	p = 0.0596
**C) P7 pTrkB/TrkB ratio**	**Naïve**	**+PB**	**0.5 mg/kg ANA12 + PB**	**2.5 mg/kg ANA12 + PB**	**5 mg/kg ANA12 + PB**
L pTrkB/TrkB ratio	0.088 ± 0.0036	0.124 ± 0.0239	0.097 ± 0.0129	0.068 ± 0.0102	0.064 ± 0.0061
R pTrkB/TrkB ratio	0.090 ± 0.0047	0.109 ± 0.0230	0.092 ± 0.0132	0.063 ± 0.0102	0.068 ± 0.0069
L pTrkB/TrkB ratio 1 way ANOVA F_4,49_ = 36.21; P < 0.0001*** post-hoc vs PB alone	p < 0.0001***	N/A	p < 0.0001***	p < 0.0001***	p < 0.0001***
R pTrkB/TrkB ratio 1 way ANOVA F_4,49_ = 22.03; P < 0.0001*** post-hoc vs PB alone	p = 0.0088**	N/A	p < 0.0001***	p < 0.0001***	p < 0.0001***
**D) P10 pTrkB/TrkB ratio**	**Naïve**	**+PB**	**0.5 mg/kg ANA12 + PB**	**2.5 mg/kg ANA12 + PB**	**5 mg/kg ANA12 + PB**
L pTrkB/TrkB ratio	0.068 ± 0.0001	0.059 ± 0.0065	0.064 ± 0.0049	0.057 ± 0.0033	0.058 ± 0.0046
R pTrkB/TrkB ratio	0.069 ± 0.0056	0.060 ± 0.0038	0.069 ± 0.0048	0.049 ± 0.0014	0.051 ± 0.0074
L pTrkB/TrkB ratio1 way ANOVA: F_4,30_ = 6.854; P = 0.0005*** post-hoc vs PB Alone	p = 0.0055**	N/A	p = 0.4971	p > 0.9999	p > 0.9999
R pTrkB/TrkB ratio1 way ANOVA: F_4,30_ = 31.99; P < 0.0001*** post-hoc vs PB Alone	p = 0.0027**	N/A	p = 0.0051**	p = 0.0008***	p = 0.0025**
**E) P7 pPLCγ/PLCγ ratio**	**Naïve**	**+PB**	**0.5 mg/kg ANA12 + PB**	**2.5 mg/kg ANA12 + PB**	**5 mg/kg ANA12 + PB**
L pPLCγ/PLCγ ratio	0.032 ± 0.0051	0.094 ± 0.0208	0.068 ± 0.0228	0.055 ± 0.0168	0.024 ± 0.0045
R pPLCγ/PLCγ ratio	0.032 ± 0.0036	0.094 ± 0.0198	0.073 ± 0.0243	0.051 ± 0.0142	0.027 ± 0.0045
L pPLCγ/PLCγ ratio 1 way ANOVA F_4,56_ = 34.95; P < 0.0001*** post hoc vs PB alone	p < 0.0001***	N/A	p = 0.0028**	p < 0.0001***	p < 0.0001***
R pPLCγ/PLCγ ratio 1 way ANOVA F_4,56_ = 37.38; P < 0.0001*** post hoc vs PB alone	p < 0.0001***	N/A	p = 0.0326*	p < 0.0001***	p < 0.0001***
**F) P10 pPLCγ/PLCγ ratio**	**Naïve**	**+PB**	**0.5 mg/kg ANA12 + PB**	**2.5 mg/kg ANA12 + PB**	**5 mg/kg ANA12 + PB**
L pPLCγ/PLCγ ratio	0.008 ± 0.0001	0.012 ± 0.0014	0.010 ± 0.0028	0.011 ± 0.0029	0.008 ± 0.0025
R pPLCγ/PLCγ ratio	0.008 ± 0.0004	0.10 ± 0.0012	0.012 ± 0.0032	0.12 ± 0.0040	0.009 ± 0.0023
L pPLCγ/PLCγ ratio 1 way ANOVA: F_4,28_ = 3.653; P = 0.0162* post-hoc vs PB Alone	p = 0.1707	N/A	p > 0.9999	p > 0.9999	p = 0.0938
R pPLCγ/PLCγ ratio 1 way ANOVA: F_4,28_ = 965; P < 0.001*** post-hoc vs PB Alone	p < 0.0001***	N/A	p > 0.9999	p > 0.9999	p > 0.9999
**I) P7 vGLUT**	**Naïve**	**+PB**	**0.5 mg/kg ANA12 + PB**	**2.5 mg/kg ANA12 + PB**	**5 mg/kg ANA12 + PB**
L vGLUT	0.469 ± 0.0505	0.274 ± 0.0256	0.585 ± 0.0486	0.616 ± 0.0335	0.478 ± 0.0547
R vGLUT	0.475 ± 0.0494	0.297 ± 0.0309	0.589 ± 0.0470	0.612 ± 0.0486	0.495 ± 0.0309
L vGLUT Independent t-test vs PB alone	t_19.549_ = −3.435 p = 0.003**	N/A	t _21.013_ = −5.655 p ≤ 0.001***	t_17_ = −7.573 p ≤ 0.001***	t_16.484_ = −3.368 p = 0.004**
R vGLUT Independent t-test vs PB alone	t_21_ = −2.481 p = 0.022*	N/A	t_22_ = −4.141 p ≤ 0.001***	t_17_ = −5.008 p ≤ 0.001***	t_17.426_ = −3.040 p = 0.007**
**J) P10 vGLUT**	**Naïve**	**+PB**	**0.5 mg/kg ANA12 + PB**	**2.5 mg/kg ANA12 + PB**	**5 mg/kg ANA12 + PB**
L vGLUT	0.533 ± 0.0059	0.375 ± 0.01164	0.573 ± 0.0020	0.539 ± 0.0166	0.707 ± 0.0116
R vGLUT	0.538 ± 0.01410	0.317 ± 0.0166	0.553 ± 0.0469	0.527 ± 0.0239	0.672 ± 0.0577
L vGLUT Independent t-test vs PB alone	t_11_ = −10.117 p < 0.001***	N/A	t_12_ = −14.482 p ≤ 0.001***	t_12_ = −12.030 p ≤ 0.001***	t_11_ = −11.381 p ≤ 0.001***
R vGLUT Independent t-test vs PB alone	t_11_ = −9.216 p < 0.001***	N/A	t_6.252_ = −4.750 p = 0.003***	t_12_ = −7.485 p ≤ 0.001***	t_11_ = −7.229 p = 0.002***

Previously, 3 h EEG data of ANA12 related seizure suppression and the corresponding 3 h post-ligation WB data^38^ (Suppl. Figure 6) demonstrated the acute role of ANA12 + PB in rescuing post-ischemic KCC2 degradation. To look at the very early role of ANA12 in the rescue of both PB-refractoriness and post-ischemic KCC2 and pKCC2 degradation in the model before PB was administered (i.e.; 1 h post-ligation) we evaluated WB data at the 1 h time-point (i.e., time point before PB administration). Data showed that ANA12 (5 mg/kg, i.e.; dose with highest anti-seizure efficacy) rescued both KCC2 and pKCC2 at 1 h (Suppl. Figure 2). These data indicate that reversal of refractoriness by ANA12 is dependent on the rescue of the early and immediate prevention of ischemia induced KCC2 hypofunction at P7.

### ANA12 rescued P10 post-ischemic KCC2 downregulation only at 5 mg/kg

24 h post-P10 ischemia, KCC2 expression was not significantly different within any treatment group (4E & F). Between groups, PB alone had significantly less contralateral KCC2 expression than naïve controls, therefore failing to rescue KCC2 expression [contralateral KCC2 expression = 73.9 ± 9.6% (*F*_4,33_ = 3.822 P = 0.012, one-way ANOVA; post-hoc, p = 0.036; vs naïve control); Fig. 4E and G]. 5 mg/kg ANA12 significantly rescued KCC2 degradation [% contralateral KCC2: 108.9 ± 7.1% (*F*_4,33_ = 3.822 P = 0.012, one-way ANOVA; post-hoc, ANA12 5 mg/kg: p = 0.018; vs. PB; Fig. 4E and G]. KCC2 downregulation was rescued by all three doses of ANA12 + PB, but there were no significant differences between doses.

24 h post-P10 ischemia, post-ischemic pKCC2 was evaluated (Fig. 4E–G). Within treatment groups, only 0.5 mg/kg of ANA12 + PB had significantly lower pKCC2 in the right hemisphere compared to the left (*t*_6_ = 2.543 p = 0.044, paired *t*-test; Fig. 4E,F). Between groups, percent contralateral pKCC2 was not significantly down-regulated in any treatment group, including PB alone, when compared to naïve controls [PB alone: % contralateral pKCC2 = 81.6 ± 11.3%, *t*_14_ = 1.099 p = 0.290, independent *t*-test; Fig. 4E and G]. There were no significant differences in ratios of pKCC2 to total KCC2 ratios between all treatment groups (Fig. 4E and H). Neither ischemia nor ANA12 significantly modulated pKCC2/KCC2 ratios, in sharp contrast to P7 (Fig. 4H; for stats, see Table 3B).

### ANA12 significantly reversed P7 ischemia induced TrkB pathway activation

**Figure 5:**
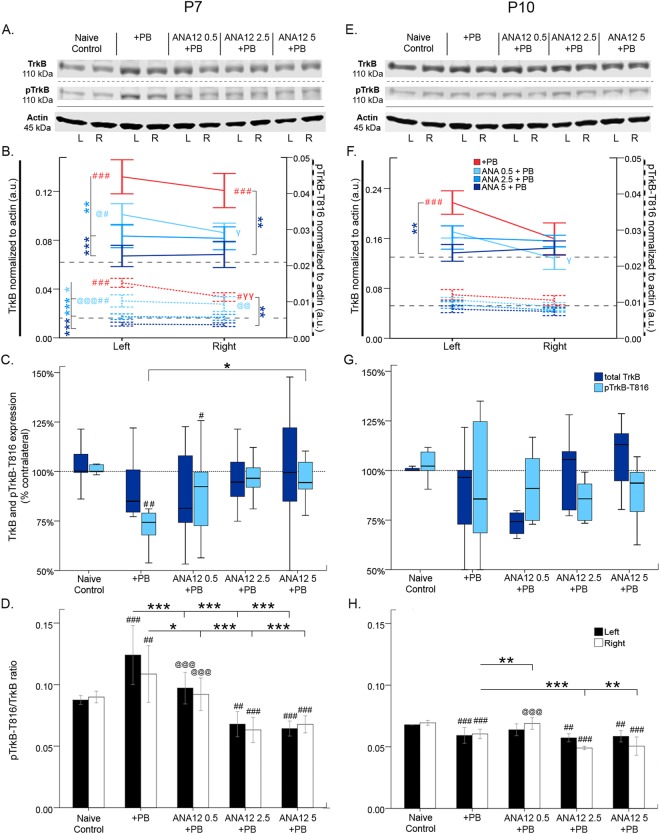
TrkB and pTrkB-T816 expression 24 h post-ischemia. (**A**) Representative Western blots showing TrkB and pTrkB expression at P7. The blots presented for each group were cropped from separate gels for improvement in clarity and conciseness of presentation. Vertical solid black lines delineate the separate blots between groups, while horizontal dotted black lines delineate separate proteins run on different gels for the same sample. All gels were run in the same experimental conditions (see methods for details). (Full-length blots presented in Supplementary Figure 5). (**B**) Quantification of blots shown in A. TrkB is upregulated by ischemia and downregulated by each dose of ANA12 + PB. (**C**) Percent contralateral expression of TrkB and pTrkB. TrkB percent contralateral is unchanged; pTrkB percent contralateral is downregulated by ischemia and rescued by 5 mg/kg of ANA12 + PB. (**D**) TrkB to pTrkB ratio. pTrkB ratio is upregulated by ischemia and rescued in a dose-dependent manner by ANA12 + PB. (**E**) Western blots showing TrkB and pTrkB expression at P10. (**F**) Quantification of blots shown in E. TrkB and pTrkB are unaffected by ischemia at P10. (**G**) TrkB and pTrkB percent contralateral. Both TrkB and pTrkB were unchanged by ischemia. (**H**) TrkB to pTrkB ratio. Significance to PB alone was done using One-way ANOVA; *P < 0.05, **P < 0.01, ***P < 0.001. Significance to naïve control: ^#^P < 0.05, ^##^P < 0.01, ^###^P < 0.001. Significance to ANA12 5 + PB: ^@^P < 0.05, ^@@^P < 0.01, ^@@@^P < 0.001. Ipsilateral versus contralateral: ^γ^*P* < 0.05, ^γγ^*P* < 0.01, ^γγγ^*P* < 0.001.

24 h post-P7 ischemia, the effect of ANA12 + PB on the TrkB pathway was investigated (Fig. 5A–D). Ischemia related insults are associated with BDNF-TrkB pathway activation^26^. Ischemic insult at P7 significantly increased TrkB and pTrkB-T816 expression bilaterally (Fig. 5A,B). However, pTrkB expression was significantly lower in the right ischemic hemisphere compared to the left (PB alone: *t*_6_ = 5.118 p = 0.002, paired *t*-test Fig. 5A,B). ANA12 + PB significantly reversed this TrkB/pTrkB upregulation at all doses (Fig. 5A,B). PB alone and 0.5 mg/kg of ANA12 + PB both had significant downregulation of percent contralateral pTrkB expression compared to naïve controls [*F*_4,51_ = 5.865 P = 0.001, one-way ANOVA; post-hoc, PB alone = 74.1 ± 5.0% (p = 0.001); ANA12 0.5 mg/kg = 85.0 ± 7.6% (p = 0.021); Fig. 5A and C]. The 5 mg/kg of ANA12 + PB group had significant rescue of percent contralateral pTrkB expression [% contralateral pTrkB = 96.4 ± 2.9% (*F*_4,51_ = 5.865 P = 0.001, one-way ANOVA; post-hoc, p = 0.038, vs. PB); Fig. 5A and C]. Only the highest dose, 5 mg/kg of ANA12 + PB, was efficacious in rescue of ischemia-induced downregulation of percent contralateral pTrkB, but had no effect on total TrkB.

pTrkB/TrKB ratio was significantly increased by ischemia; ANA12 + PB rescued this ratio in a dose-dependent manner (Fig. 5D; for stats, see Table 3C).

### TrkB pathway was not significantly modulated at P10 by ischemia or ANA12

24 h post-P10 ischemia, the effect of ANA12 + PB on the TrkB pathway was investigated (Fig. 5E–H). At P10, ischemic insult also resulted in a similar upregulation of TrkB, but was only significant in the left hemisphere (TrkB: Left-*F*_4,32_ = 6.782 P ≤ 0.001, one-way ANOVA; post-hoc, p ≤ 0.001; vs naïve control; Fig. 5E,F). No significant upregulation of pTrkB was detected at P10; (Fig. 5F). Only 5 mg/kg of ANA12 + PB significantly rescued ischemia induced upregulation of TrkB in the left hemisphere compared to PB alone, which was not detected for pTrkB (TrkB: Left-*F*_4,32_ = 6.782 P ≤ 0.001, one-way ANOVA; post-hoc, p = 0.004; Fig. 5E,F). Compared to PB alone, ANA12 + PB did not significantly alter percent contralateral TrkB/pTrkB expression (Fig. 5E,G). Neither ischemia nor any dose of ANA12 + PB significantly modulated TrkB or pTrkB in the right ischemic hemisphere at P10. pTrkB/TrkB ratios at P10 following ischemia + PB were modulated differently compared to P7 (i.e.; ratio was lower rather than higher compared to age-matched controls, Fig. 5 compare D vs. H). The higher doses of ANA12 + PB significantly further lowered pTrkB/TrkB ratios at P10 (Fig. 5H; for stats, see Table 3D) except with 0.5 mg/kg. This indicated that the TrkB-pathway did not play a significant role in post-ischemic seizure generation when PB remained efficacious and no refractoriness was detected.

### ANA12 reversed P7 ischemia induced TrkB phosphorylation of PLCy1

**Figure 6:**
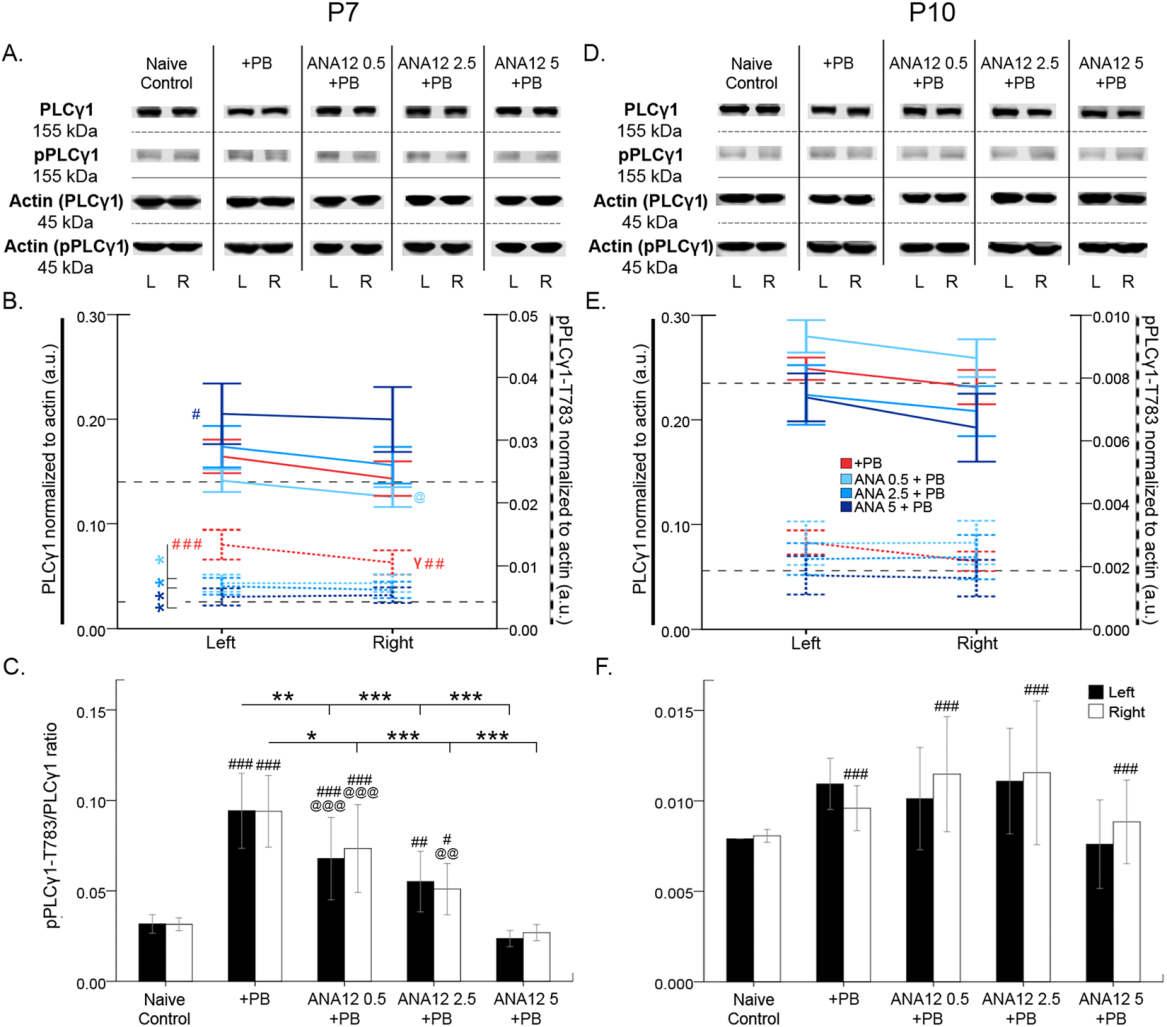
PLCγ1 and pPLCγ1-T783 expression 24 h post-ischemia. (**A**) Representative Western blots showing PLCγ1 and pPLCγ1 expression at P7. The blots presented for each group were cropped from separate gels for improvement in clarity and conciseness of presentation. Vertical solid black lines delineate the separate blots between groups, while horizontal dotted black lines delineate separate proteins run on different gels for the same sample. All gels were run in the same experimental conditions (see methods for details). (Full-length blots of each tested presented in Supplementary Figure 6). (**B**) Quantification of Western blots shown in A. PLCγ1 is not modulated by ischemia. pPLCγ1 is upregulated by ischemia and rescued in a dose-dependent manner by ANA12 + PB. (**C**) PLCγ1 to pPLCγ1 ratio. pPLCγ1 ratio is increased by ischemia and rescued by 5 mg/kg of ANA12 + PB. (**D**) Western blots showing PLCγ1 and pPLCγ1 expression at P10. (**E**) Quantification of Western blots shown in D. At P10, PLCγ1 and pPLCγ1 are not modulated by ischemia. (**F**) PLCγ1 to pPLCγ1 ratio at P10. Significance to PB: *P < 0.05, **P < 0.01, ***P < 0.001. Significance to naïve control: ^#^P < 0.05, ^##^P < 0.01, ^###^P < 0.001. Ipsilateral vs. contralateral: ^γ^*P* < 0.05, ^γγ^*P* < 0.01, ^γγγ^*P* < 0.001.

24 h post-P7 ischemia, the effect of ANA12 + PB on TrkB associated PLCγ1 expression was investigated (Fig. 6A–C). PLCγ1 activation by TrkB has been shown to promote epileptogenesis^40,41^ and phosphorylation of PLCγ1 has been shown to downregulate KCC2^26,42^. Ischemic insult at P7 did not modulate PLCγ1, but significantly increased pPLCγ1-T783 bilaterally (Fig. 6A and B). However, pPLCγ1 expression was significantly lower in the right ischemic hemisphere compared to the left (*t*_7_ = 3.531 p = 0.010, paired *t*-test; Fig. 6A and B). ANA12 + PB significantly reversed this pPLCγ1 upregulation in the left hemisphere at all doses (*F*_4,57_ = 6.022 P ≤ 0.001, one-way ANOVA; post-hoc, ANA12 0.5 mg/kg: p = 0.035, ANA12 2.5 mg/kg: p = 0.027, ANA12 5 mg/kg: p = 0.004; vs PB; one-way ANOVA, Fig. 6A and B).

Ischemia raised pPLCγ/PLCγ ratio bilaterally at P7. ANA12 + PB rescued this upregulation in a dose-dependent manner (Fig. 6C; for stats, see Table 3E).

### PLCγ1 phosphorylation not significantly modulated at P10 by ANA12

24 h post-P10 ischemia, the effect of ANA12 + PB on TrkB associated PLCγ1 expression was investigated (Fig. 6D–F). PLCγ1and pPLCγ1 expression were not significantly different between all treatment groups. Neither ischemia nor any dose of ANA12 + PB significantly modulated PLCγ1 or pPLCγ1at P10. pPLCγ1/PLCγ1 ratios however were significantly higher in the right ischemic hemisphere at P10, but ANA12 failed to modulate them (Fig. 6F; for stats, see Table 3F).

### Post-ischemic vGLUT1 depletion rescued by ANA12 + PB

**Figure 7:**
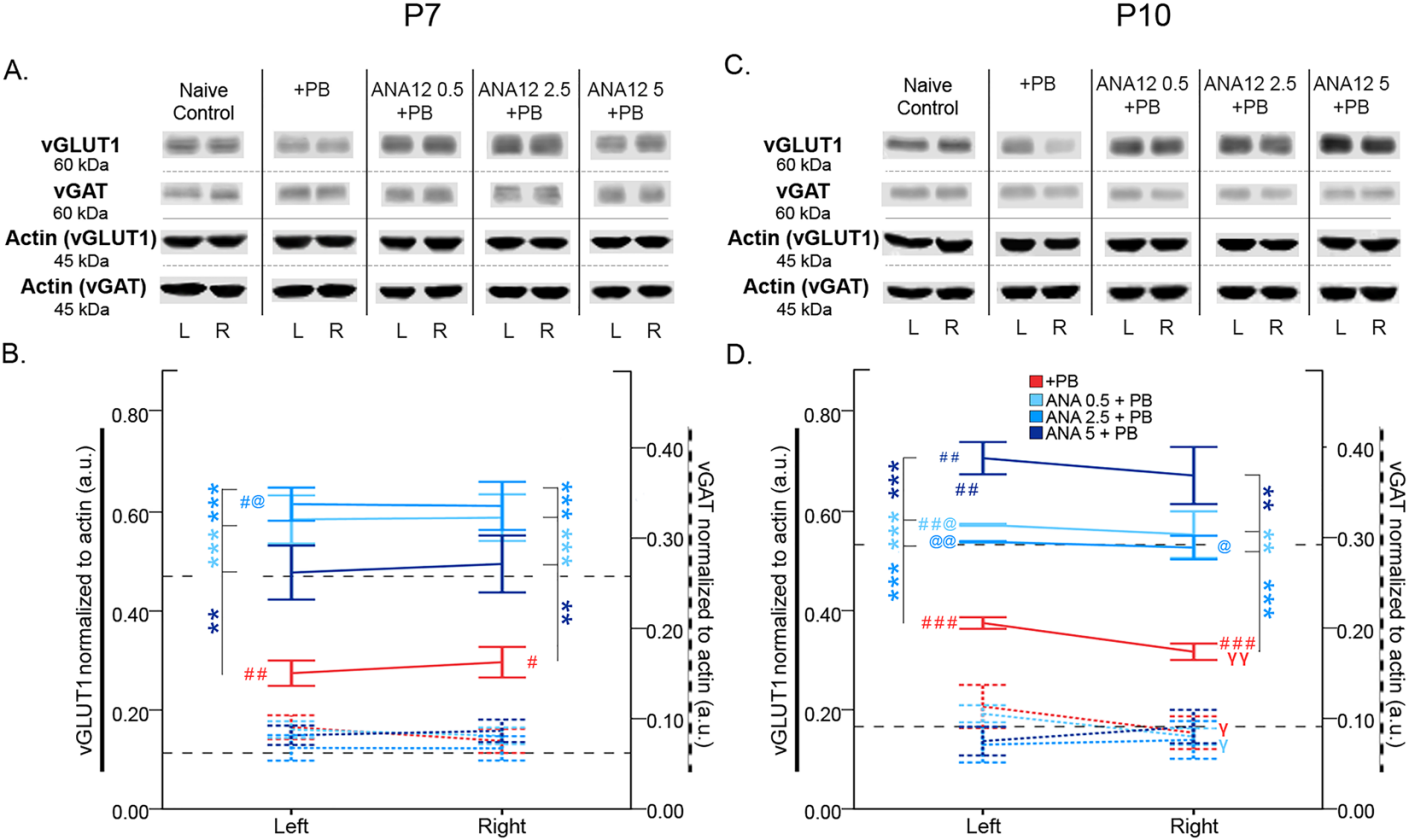
vGLUT1 vs. vGAT expression 24 h post-ischemia. (**A**) Representative Western blots showing vGLUT1 and vGAT expression at P7. The blots presented for each group were cropped from separate gels for improvement in clarity and conciseness of presentation. Vertical solid black lines delineate the separate blots between groups, while horizontal dotted black lines delineate separate proteins run on different gels for the same sample. All gels were run in the same experimental conditions (see methods for details). (Full-length blots of each tested presented in Supplementary Figure 7). (**B**) Quantification of Western blots shown in A. vGLUT1 is downregulated by ischemia and rescued by all doses of ANA12 + PB in a dose-dependent manner. vGAT is not modulated by ischemia. (**C**) Representative Western blots showing vGLUT1 and vGAT expression at P10. (**D**) Quantification of blots shown in C. vGLUT1 is downregulated by ischemia and rescued in a dose-dependent manner by ANA12 + PB. vGAT is unaffected by ischemia. Significance to PB alone: *P < 0.05, **P < 0.01, ***P < 0.001. Significance to naïve control: ^#^P < 0.05, ^##^P < 0.01, ^###^P < 0.001. Significance to 5 mg/kg of ANA12 + PB: ^@^P < 0.05, ^@@^P < 0.01, ^@@@^P < 0.001. Ipsilateral to contralateral: ^γ^*P* < 0.05, ^γγ^*P* < 0.01, ^γγγ^*P* < 0.001.

Seizures can modulate glutamate output by changing the transmitter content of synaptic vesicles^43^. In this study, ischemia significantly depleted vGLUT1 24 h post-ischemia (Left-*t*_19.549_ = −3.435 p = 0.003, Right-*t*_21_ = −2.481 p = 0.022 respectively; independent *t*-test vs. naïve control; Fig. 7A and B). ANA12 + PB significantly rescued vGLUT1 depletion at P7 at all doses tested (Fig. 7A and B; for stats see Table 3). In contrast, vGAT expression was not significantly modulated by ischemia or ANA12 + PB.

At P10, significant post-ischemic vGLUT1 depletion was rescued by ANA12 + PB in a dose-dependent manner Fig. 7E and F; for stats see Table 3). This dose-dependent rescue of vGLUT1 was not evident at P7. At P10, vGAT was significantly depleted in the right hemisphere compared to left in the PB alone group (vGAT: *t*_6_ = 3.306 p = 0.016; paired *t*-test; Fig. 7E and F). These data indicate vGLUT1 and vGAT were differentially modulated by ischemic insult and ANA12 + PB significantly rescued vGLUT1 depletion at both P7 and P10. At P7 and P10 no significant activation of CREB (pCREB-S133) was detected at 24 h post-ischemia, compared to naïve controls (Figure supplement 3A–D).

### KCC2 hypofunction and TrkB activation were age-dependent

**Figure 8:**
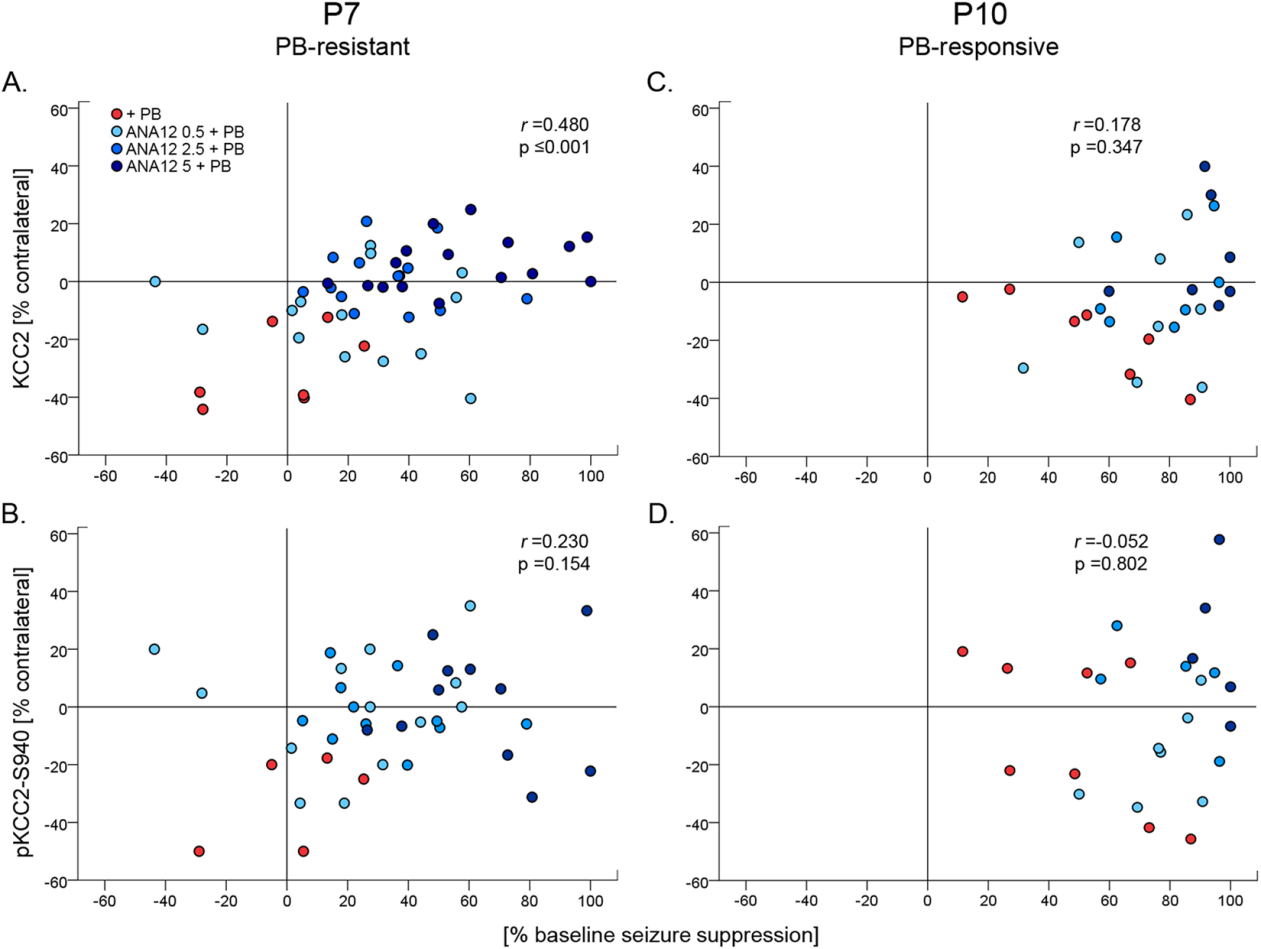
Correlation of KCC2 and pKCC2 expression vs. Seizure suppression. (**A**) KCC2 and seizure suppression showed a significant positive correlation at P7, when seizures are PB-resistant. (**B**) pKCC2 and seizure suppression were positively correlated, but not significant at P7. (**C**,**D**) KCC2 and pKCC2 show no correlation at P10, when seizures are PB-responsive.

Analysis of KCC2 post-ischemic expression indicated unilateral degradation of KCC2.Previous data has shown early degradation of KCC2 (i.e. 3 h) following ischemia in this model^25,38^. To evaluate the dose-dependent effect of ANA12 on this early degradation, the percent contralateral expression of KCC2 to seizure suppression was evaluated. Seizure suppression significantly correlated with the rescue of post-ischemic KCC2 degradation at P7, but not at P10. (r_(49)_ = 0.480, p ≤ 0.001; r_(28)_ = 0.178, p = 0.347 respectively; Spearman’s test; Fig. 8A and C). pKCC2 had no significant correlations with seizure suppression at P7 or P10 (pKCC2 vs. seizure suppression: r_(38)_ = 0.154, p = 0.230; r_(24)_ = −0.052, p = 0.802; respectively, Spearman’s test; Fig. 8B and D). At both ages, correlations between KCC2 and pKCC2 were not significant bilaterally. Although pKCC2 is known to play a significant role in KCC2 membrane stability, data showed that when all treatment groups are pooled together, only total KCC2 was significantly associated with seizure suppression outcomes in this model at P7 but not at P10.

**Figure 9:**
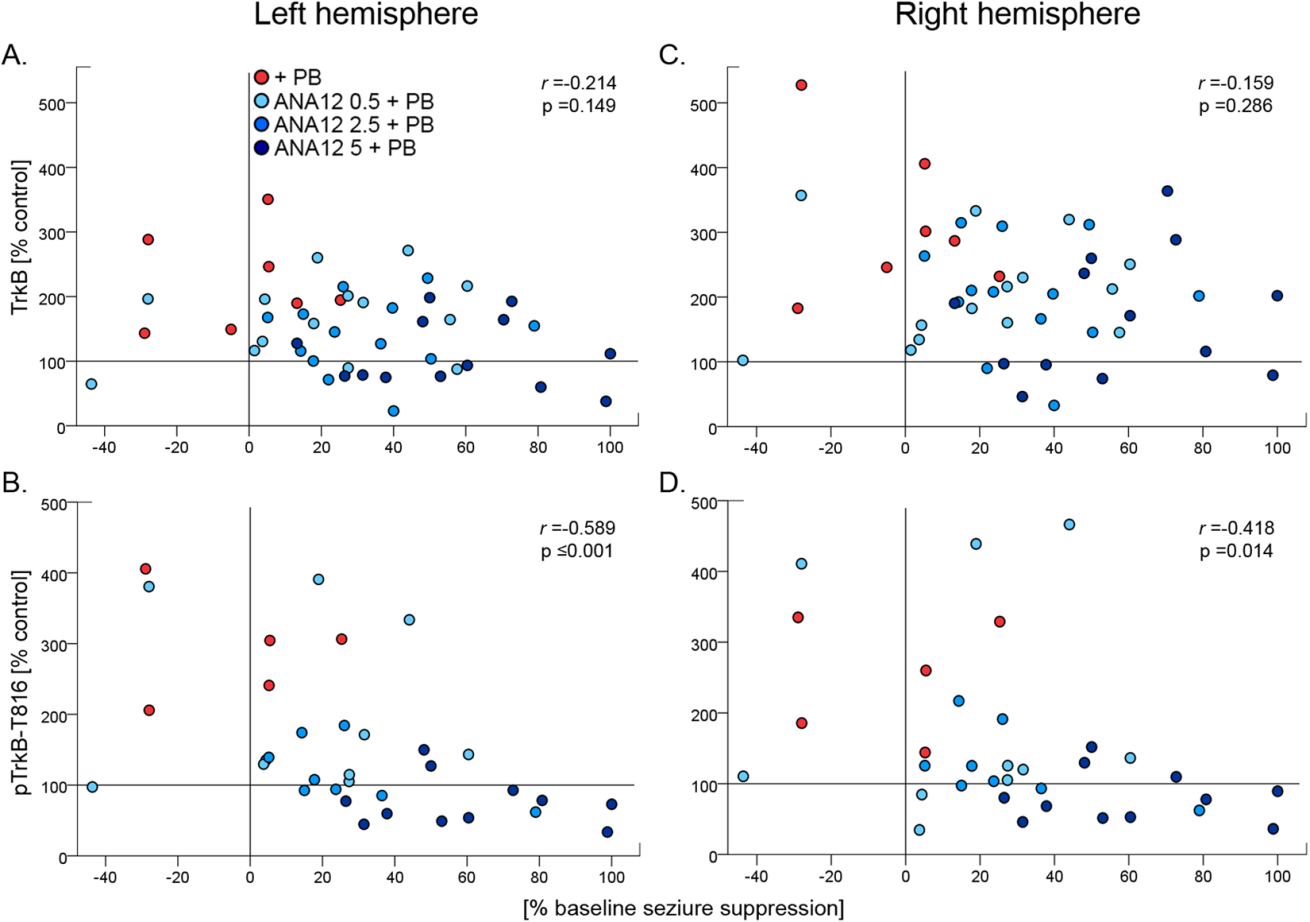
Correlation of left and right hemisphere TrkB and pTrkB-T816 vs. Seizure suppression at P7. TrkB expressions in bilateral hemispheres were not significantly correlated with seizure suppression. However, pTrkB in bilateral hemispheres had a significant negative correlation with seizure suppression (p-values calculated by Spearman’s test).

In contrast, post-ischemic TrkB expression indicated a bilateral increase to unilateral ischemia. Therefore, bilateral TrkB expression and its correlation to seizure suppression were evaluated (Fig. 9A–D). TrkB expressions in bilateral hemispheres were not significantly correlated with seizure suppression at neither P7 nor P10. At P7, pTrkB in bilateral hemispheres had a significant negative correlation with seizure suppression (left hemisphere pTrkB vs. seizure suppression: r_(32)_ = −0.589, p ≤ 0.001; right hemisphere pTrkB vs. seizure suppression: r_(32)_ = −0.418, p = 0.014, Spearman’s test;), neither of which were significant at P10. Unlike KCC2, which was differentially downregulated in the right hemisphere and had no correlation to pKCC2, pTrkB to TrkB had a significant positive correlation in bilateral hemispheres at both ages (pTrkB vs. TrkB, P7: Left-r_(52)_ = 0.704, p ≤ 0.001; Right-r_(52)_ = 0.615, p ≤ 0.001; P10: Left-r_(33)_ = 0.567, p ≤ 0.001; Right-r_(33)_ = 0.437, p = 0.009; Spearman’s test). PLCγ1 and pPLCγ1 were not significantly correlated with seizure suppression, nor to each other, at P7 or P10. Therefore, the activation of the TrkB pathway by phosphorylation of T816 following ischemia was age dependent.

### Age- and sex-dependent differences in ANA12 + PB dose-dependent efficacy

Males have higher seizure susceptibilities both in animal models and in the clinic^25,44^. Therefore, ANA12 + PB may act differentially by sex. Both 0.5 and 2.5 mg/kg of ANA12 + PB failed as an adjunct to PB in males, but had a significant effect in females [% seizure suppression: 0.5 mg/kg and 2.5 mg/kg of ANA12 + PB (females: *F*_3,39_ = 17.627 P ≤ 0.001, males: *F*_3,44_ = 8.808 P ≤ 0.001, one-way ANOVA; post-hoc, ANA12 0.5 mg/kg: females, p = 0.010, and males, p = 1.000; ANA12 2.5 mg/kg: females, p ≤ 0.001, and males, p = 0.078; vs. respective PB alone)]. Both sexes responded efficaciously to 5 mg/kg of ANA12 + PB. Males responded less efficaciously and required higher doses of ANA12 + PB than females at P7. At P10, there was no significant difference between sexes.

To further investigate the sex-dependent efficacy of ANA12 at P7, we analyzed the protein data by sex. At P7, ischemia significantly upregulated TrkB in the ischemic hemisphere in males but not females (Males: TrkB: Right, *F*_4,29_ = 6.693 P = 0.007, one-way ANOVA; post-hoc, p = 0.020; vs. naïve control group). TrkB activation has been linked to KCC2 downregulation^26,42^. These data may explain the sex-differential activity of ANA12 + PB; however, further studies are needed. At P10, the age when PB was effective, there were no significant sex differences in KCC2 or TrkB expression.

P7 baseline seizure burdens in the PB alone group were significantly higher than P10, replicating age-dependent seizure susceptibility (*t*_33_ = 3.619 p = 0.001; P7 vs P10; independent t-test; Kang *et al*.^25^). At P7, naïve controls had higher levels of TrkB and pTrkB than at P10 (TrkB: Left-*t*_17.000_ = −16.387 p ≤ 0.001, Right-*t*_24_ = −9.802 p ≤ 0.001; pTrkB: Left-*t*_22.000_ = −19.710 p ≤ 0.001, Right-*t*_29_ = −10.331 p ≤ 0.001; independent *t*-test). P7 pups may experience greater TrkB activation and therefore worse KCC2 downregulation after ischemic insult. After ischemia, pKCC2 to KCC2 ratio is significantly downregulated in P7 pups compared to P10 (Left-*t*_9.089_ = −2.688 p = 0.025, Right-*t*_8.336_ = −2.625 p = 0.029, independent *t*-test). These data may explain why ANA12, a small-molecule TrkB antagonist, works in a dose-dependent manner at P7 but not P10.

## Discussion

This study has several key findings: 1. Small-molecule TrkB antagonist, ANA12, reversed post-ischemic PB-resistant seizures in a dose-dependent manner; 2. Rescue of PB-resistance was associated with a dose-dependent rescue of KCC2 downregulation; 3. The pKCC2-S940 site played a significant role in the rescue of KCC2 membrane stability associated with ANA12 + PB anti-seizure efficacy; 4. The TrkB pathway was significantly activated following neonatal ischemia at P7 with upregulation of both pTrkB and its downstream activation site pPLCγ1, each were rescued to control levels; 5. Reversal of ischemia induced pTrkB activation correlated significantly with percent seizure suppression at P7, 6.TrkB pathway activation at P10 via pTrkB-T816 was not significant, nor was there PB-resistance at that age. 7. Ischemia induced vGLUT1 depletion following insult which was significantly rescued by ANA12 + PB, in contrast, vGAT showed no significant modulation.

Ischemic insults leads to rapid increase in BDNF, and BDNF binding to the TrkB receptor is responsible for the pathophysiological downregulation of KCC2^26–28,42^. K252a, a TrkB receptor antagonist, was shown to produce a significant increase in KCC2 levels in organotypic hippocampal slice cultures^26^. Another well studied area is the intrinsic functional modulation of KCC2 by phosphorylation at various residues^23,45^. The dephosphorylation of the pKCC2-S940 site triggers the internalization of membrane bound KCC2, thus reducing surface expression and hence function^23,46^. The acute, specific and transient post-ischemic inhibition of the TrkB receptor with ANA12 can help prevent acute KCC2 downregulation^25,38^. However, the age-, sex- and dose-dependence underlying the mechanism for TrkB mediated KCC2 downregulation in neonates are unknown.

KCC2, in addition to its role in maintaining the Cl- transmembrane gradient, has also been shown to play an independent role in cell-survival, synaptogenesis, and AMPA receptor insertion at excitatory synapses^47–49^. KCC2 modulation may be a beneficial pharmacological target for excitotoxic insults during development that result in increased susceptibilities to long-term sequelae like epilepsy^50^, schizophrenia^51^, and disorders of dendritic spine formation^52^.

KCC2 is highly expressed at excitatory synapses, and is essential for glutamatergic synapse development and function. The suppression of KCC2 activity reduces the localization of GluR1 composed AMPA receptors at the synapse, and subsequently reduces the amplitude of EPSCs^53^. Increased glutamatergic signaling also increases KCC2 membrane dynamics at excitatory synapses, as NMDA activation leads to Ca^2+^ dependent dephosphorylation of KCC2-S940^54^. Therefore, in addition to being the chief neuronal chloride extruder, KCC2 also curbs hyperexcitability by attenuating the response to glutamatergic signaling at excitatory synapses.

In contrast, hyperpolarizing GABA_A_ signaling stabilizes KCC2 at inhibitory synapses^55^. Other pathways known to modulate KCC2 function include WNK1, a chloride sensitive kinase, that is activated by low intracellular Cl^−^ levels, and phosphorylates KCC2 at T906 and T1007^55^. Wright *et al*. 2017 have demonstrated that metabotropic GABA_B_ receptor activation lead to clathrin-mediated endocytosis of KCC2^56^. The calpain mediated pathway activated by prenatal hypoxia-ischemia can also degrade KCC2, erythropoietin has demonstrated an ability to rescue this degradation^57^. Therefore, KCC2 hypofunction in neonatal ischemia may alter intrinsic excitability of neurons at both excitatory and inhibitory synapses. Further studies are required to determine KCC2’s role within specific cell types, and their associated injury from hypoxic-ischemic insults.

Previous studies have demonstrated that a history of seizures prior to PB administration can determine PB efficacy^58^. Nardou *et al*. showed PB was efficacious in stopping early seizures but aggravated established ones. Therefore, administration of GABA agonists like PB could result in seizure aggravation in model systems where KCC2 function has been compromised. Recently, Chen *et al*. 2018 has demonstrated KCC2 downregulation preceding and facilitating seizures^59^. This supports ANA12’s significance as a potential therapeutic to prevent KCC2 downregulation, and prevent an increased propensity for seizures due to KCC2’s hypofunction.

Glutamatergic neurotransmission is highly dependent upon the function of vGLUTs to transport glutamate into synaptic vesicles. One of the features of VGLUT function is its Cl^−^ dependence, which is not fully understood. Cl^−^ modulates vGLUT1, and thus modulates glutamate transport into vesicles^60^. The allosteric activation of vGLUT1 and vGLUT2 is inhibited by ketone bodies, consistent with clinical outcomes reported for the ketogenic diet used to treat children with drug-resistant epilepsies^61,62^. This suggests a possible role of intracellular chloride concentrations as a second messenger in the presynaptic terminal, modulating vGLUT1.Further studies are required to understand the *in vivo* intracellular Cl^−^ concentrations at the presynaptic terminal, how vGLUT1 modulation affects systemic neurotransmission, and the direct effects of seizures on both presynaptic intracellular chloride levels and vGLUT1. In this study, ischemia induced seizures depleted vGLUT1 but had no effect on vGAT, consistent with previous reports^35^. ANA12 + PB was able to rescue this depletion of VGLUT1 at all of the doses tested, supporting that vGLUT depletion is mediated by seizures.

This study provides novel insights into the unknown developmental influence on the TrkB pathway. TrkB pathway modulation played a significant role in the dose-dependent anti-seizure efficacy of ANA12 for PB-resistant seizures at P7, but not for the PB-responsive seizures at P10. In contrast, ANA12 played a significant role in the rescue of post-ischemic dephosphorylation of pKCC2-S940 at both P7 and P10. The age-dependent efficacy of PB for neonatal ischemic seizures may underlie the developmental switch from GABA acting as a depolarizing agent in immature brains, to its hyperpolarizing function in mature brains^14^ in this CD-1 mouse model^25^. ANA12 is the first novel low-molecular weight TrkB antagonist^36^ that crosses the blood brain barrier. It binds to the extracellular domain of TrkB and prevents BDNF-induced TrkB activation, but does not prevent the biological action of BDNF on NGF or NT-3 on TrkA- and TrkC-expressing cells. ANA12 has been shown to inhibit TrkB in the brain after systemic administration without any significant adverse effects, even with multiple dosing protocols in adult mice^36^.

This study demonstrates that power is not a reliable proxy for seizures, especially when recording from free moving subjects such as seizing pups. In this study, electrode sites differ slightly in placement and therefore no between subject power analysis would be meaningful. Additionally, repetitive seizures change in amplitude and frequency as a function of time; this is especially true for ischemic seizures^4,7,63^. Therefore, EEG power has an abbreviated role in acute seizure analysis (i.e. high power or lower power seizure) and is only meaningful within a subject, and not between subjects. Our findings are similar to reports demonstrating low sensitivity to seizure detection algorithms that rely on power, yet high accuracy on algorithms that utilize time and frequency characteristics^64^.

The TrkB pathway plays a critical role in the post-ischemic emergence of refractory seizures. This characterized CD1 mouse model of PB-resistant seizures was shown to be age-dependent^25^ and this study showed that the TrkB pathway modulation was also significantly age-dependent. KCC2 downregulation occurred only in the ischemic hemisphere indicating a direct effect of ischemia; in contrast pKCC2 downregulation occurred bilaterally indicating that this may occur due to repetitive seizures. The pKCC2-S940 phosphorylation site also seemed to play a role at P7 and not at P10, indicating that its role in KCC2 hypofunction was age-dependent which may be associated with the seizure severity and PB-refractoriness that occurs at P7. It is not clear whether the rescue of pKCC2-S940 with ANA12 is a direct effect of ANA12 interacting with TrkB or an indirect effect by preventing TrkB mediated KCC2 downregulation. However, KCC2 S940A mutants show functional deficits following transient exposure to glutamate, suggesting that the S940 site plays an important role in KCC2 activity during excitotoxic conditions, in contrast to basal conditions^45^. Future studies with TrkB mutant mice may help answer this question. However, the data presented here demonstrate that ANA12, a small-molecule TrkB antagonist rescues PB-refractoriness and KCC2 hypofunction by reliably and significantly reversing ischemia induced TrkB-pathway activation at P7 in a dose-dependent manner.

## Materials and Methods

All experimental procedures were conducted in compliance with guidelines by the Committee on the Ethics of Animal Experiments, Johns Hopkins University (Permit Number: A3272-01) and all protocols were approved by the Animal Care and Use of Committee (IACUC) of Johns Hopkins. All litters of CD1 mice with dams were purchased from Charles River Laboratories Inc. (Wilmington, MA.). Newly born litters of pups (n = 10) with dams were delivered at postnatal days old (P3 or P4) and allowed to acclimate. Food and water were provided ad libitum. Equal numbers of male and female pups were used in the study. Sample sizes are included in Table 1.

### Surgical procedure for ischemic insult and sub-dermal EEG electrode implantation

The surgical protocol was similar to the previously published work^25,65^. At P7 or P10, animals were subjected to permanent unilateral ligation (without transection) of the right common carotid artery using 6-0 surgisilk (Fine Science Tools, BC Canada) under isoflurane anesthesia. The outer skin was closed with 6-0 monofilament nylon (Covidien, MA), and lidocaine was applied as local anesthetic. Under continued anesthesia, animals were then implanted with 3 sub-dermal EEG scalp electrodes: 1 recording and 1 reference overlying the bilateral parietal cortices, and 1 ground electrode overlying the rostrum. Wire electrodes made for use in humans (IVES EEG; Model # SWE-L25 –MA, IVES EEG solutions, USA) were implanted sub-dermally and fixed in position with cyanoacrylate adhesive (KrazyGlue). Pups were then allowed to recover from anesthesia over a few minutes. Pups were then tethered to a preamplifier by connecting to the sub-dermal electrodes within a recording chamber for 2 h of continuous video-EEG recording, maintained at 36 °C with isothermal pads. At the end of the recording session, sub-dermal electrodes were removed, and the pups were returned to the dam. The average duration of anesthesia for both ligation and electrode implantation surgery was 16.18 ± 4.37 min. There is a known mortality rate of ~10–20% associated with the surgical procedure of carotid-ligation and severe seizures in the model^66^. The mortality rates for the pups 24 h after surgery were n/n = 7/149 pups at P7 (5 males and 2 females) and n/n = 0/49 pups at P10, and were not significantly different, by age nor by sex (p = 0.20 and p = 0.70 respectively; Fisher’s exact test, two-tailed). Mortality rates following the surgical procedure were also not significantly different by treatment (ligated control vs. treated group; p = 0.19; Fisher’s exact test, two-tailed).

### Experimental paradigm

ANA12 (N-[2-[[(Hexahydro-2-oxo-1H-azepin-3-yl) amino] carbonyl] phenyl] benzo [b]thiophene-2-carboxamide) at doses of 0.5 mg/kg, 2.5 mg/kg, and 5 mg/kg (Sigma- Aldrich; Cat. No. SML0209) was injected intraperitoneally (IP) following carotid-ligation. The experimental paradigm is depicted in Fig. 1A. ANA12 was dissolved in 5% dimethyl sulfoxide (DMSO) and stored at −20 °C in aliquots. ANA12 dose-range was chosen based on the efficacious dose previously published in our pilot ‘proof of principle” paper^25^ and expanded based on safety studies that determined non-toxic higher doses in mice with chronic dosing protocols^36^. A previous study showed that DMSO alone did not alter seizure burdens in this model, hence a vehicle alone treatment group was not included here^38^. PB (25 mg/kg; Sigma-Aldrich; Cat. No. P5178) was dissolved in phosphate buffered saline and injected 1 h after the ANA12 injections. The 25 mg/kg PB loading dose replicated a pilot study protocol and other similar pre-clinical studies^9,25^. Recent clinical trials have also shown that when a loading dose of PB at 20 mg/kg was ineffective, a second follow-on dose of 20 mg/kg over a 12 h period^4,67^ was ineffective in curbing neonatal seizures therefore no additional doses of PB were administered here.

### *In vivo* synchronous video-EEG recording and analyses

EEG recording was acquired using Sirenia Acquisition software (v 1.6.4) with synchronous video capture (Pinnacle Technology Inc. KS, USA). Data acquisition was done with sampling rates of 400 Hz that had a pre-amplifier gain of 100 and a 0.5 Hz to 50 Hz band pass filter. Data were scored by binning EEG in 10 sec epochs. Similar to our previous study^25^, seizures were defined as electrographic ictal events that consisted of rhythmic spikes of high amplitude, diffuse peak frequency of ≥7–8 Hz (i.e.; peak frequency detected by automated spectral power analysis) lasting ≥6 seconds (i.e.; longer than half of each 10 sec epoch on the manual scoring screen within the module). Short duration burst activity lasting <6 seconds (brief runs of epileptiform discharges) were not included for seizure burden calculations similar to previous studies in the model^25^. Mean time spent seizing for 1^st^ h baseline seizure burden vs. 2^nd^ h post-PB seizure burden was quantified in seconds. Mean seizure suppression was calculated by: % seizure suppression = −100* (1^st^ h baseline seizure burden – 2^nd^ h post-PB seizure burden)/1^st^ h baseline seizure burden. Mean number of ictal events (count) and ictal durations (seconds/event) were also calculated for 1^st^ vs. 2^nd^ h.

### EEG power analysis

EEG power was generated analyzed using Sirenia Sleep software (v 1.7.10; Pinnacle Technology Inc.). The full EEG power from 0.5 to 50 Hz was acquired for every 10 second epoch after Fourier transformation. Spectral power data were binned as delta (0.5–4.0 Hz), theta (5.5–8.0 Hz), alpha (8.0–13.0 Hz), beta (13.0–30.0 Hz) and gamma (35–50 Hz). Total EEG power was calculated for the first hour (3600 seconds) and second hour of recording, post-PB injection (3600 seconds). The total EEG power for every 5 minutes (24 epochs of 300 seconds) was calculated for each 2 hour recording. From each treatment group, 6 randomly chosen P7 pups (after excluding pups with electrical noise related EEG artifacts) were used for EEG power analysis [PB alone: n = 6(4 males, 2 females); ANA12 0.5 + PB: n = 6 (3 males, 3 females); ANA12 2.5 + PB: n = 6 (4 males and 2 females); ANA12 5 + PB: (2 males and 4 females); Total N = 24].

### Western blot analysis at 24 h post-ligation

All animals for immunochemical characterizations were anesthetized with chloral hydrate (90 mg/ml; IP) before transcardial perfusion with ice cold saline. Whole fresh brains were removed, separated into left and right cerebral hemispheres, and frozen in dry ice for storage at −80 °C. Brain tissue was homogenized and suspended in TPER cell lysis buffer containing 10% protease/phosphatase inhibitor cocktail. Total protein amounts were measured using Bradford protein assay (Bio-Rad, Hercules, CA, USA) at 570 nm wavelength. 25ug per 20 uL of protein samples were run on 4–20% gradient SDS gels (Invitrogen, Gand Island, NY, USA) for 100–120 min with 130 V, and were transferred onto nitrocellulose membranes overnight at 20 V. After the transfer, the nitrocellulose membranes underwent 1 h blocking in Rockland buffer before overnight incubation with primary antibodies (supplementary Table 1): rabbit α KCC2 (1:1000, Millipore; Cat. No. 07–432), rabbit α phospho-KCC2(S940) (1:1000 Aviva Systems Biology; Cat. No. OAPC00188), mouse α TrkB (1:1000, BD Biosciences; Cat. No. 610102), rabbit α phospho-TrkB(T816) (1:500, Millipore; Cat. No. ABN1381), mouse α PLCγ1 (1:1000, Thermo Scientific; Cat. No. LF-MA0050), rabbit α phospho-PLCγ1(T783) (1:1000, Cell Signaling Technology; Cat. No. 2821 S) [1 h blocking in 1x TBS in 5% nonfat dry milk for antibodies PLCγ and pPLCγ (T783), not Rockland], guinea pig α vGLUT1 (1:5000, Synaptic Systems; Cat. No. 135 304), rabbit α vGAT(1:1000, Thermo Scientific; Cat. No. PA5-27569), rabbit α phospho-CREB(S133) (1:1000, Millipore; Cat. No. 06–519), and mouse α actin (1:10000, LI-COR Biosciences, Cat. No. 926–42214). Nitrocellulose membranes were washed with TBS-T and incubated in chemiluminescent secondaries for 1 h (1:5000 for all goat α-mouse 680LT, goat α rabbit 800CW, and donkey α guinea pig 680RD; LI-COR Biosciences). Chemiluminescent protein bands were analyzed using the Odyssey infrared imaging system 2.1 (LI-COR Biosciences). Optical density of each protein sample was normalized to their corresponding actin bands run on each lane for internal control. Mean normalized protein expression levels were calculated for respective left and right cerebral hemispheres. In this model of unilateral ischemia, the right hemisphere is ipsilateral and left hemisphere is contralateral. All proteins quantified in the ipsilateral ischemic hemispheres were normalized to contralateral non-ischemic hemispheres in the same brain to compare differential effects of ischemic injury in the model.

$$ \% \,{\rm{contralateral}}\,{\rm{expression}}\,({\rm{right}}\,{\rm{over}}\,{\rm{left}})=[{\rm{100}}\ast ({\rm{ipsilateral}}/{\rm{contralateral}}\,{\rm{protein}}\,{\rm{expression}})]$$

### Statistics

Statistical tests were performed using SPSS21 (IBM) except two-way ANOVA, which were run in Prism 7 (Graphpad). Group means of total seizure burden, number of ictal events, and ictal duration within each treatment group and between groups were compared using two-way ANOVA with Bonferroni’s post hoc correlations. Western blot data were compared between treatment groups using one-way ANOVAs and independent sample *t*-tests. Correlation analyses were performed using nonparametric comparisons (Spearman’s test, two-tail). All error bars represent ± 1 standard error of the mean. P-values less than or equal to the alpha at 0.05 (P ≤ 0.05) were considered statistically significant.

## Electronic supplementary material

Supplementary information

P7 Rescue of PB Resistance
