# Supplementary material for: Dose-dependent reversal of KCC2 hypofunction and phenobarbital-resistant neonatal seizures by ANA12
Source: Sci Rep. 2018 Aug 10;8:11987. doi: 10.1038/s41598-018-30486-7 (PMC6086916; doi:10.1038/s41598-018-30486-7)
Supplement: Supplementary file 1 — Supplementary information [file 41598_2018_30486_MOESM1_ESM.docx]

**Dose-dependent reversal of KCC2 hypofunction and phenobarbital-resistant neonatal seizures by ANA12.**

**Carter B.M.^1^; Sullivan B.J. ^1^; Landers J.R. ^1^; Kadam S.D.^1, 2*^**

Neuroscience Laboratory^1^, Hugo Moser Research Institute at Kennedy Krieger; Department of Neurology^2^, Johns Hopkins University School of Medicine; Baltimore, MD 21205

**Supplemental Information**

**Supplemental Video 1:** video EEG of rescue of PB-refractoriness by ANA12

(Attached PPT file).

**Supplemental Table 1:** Western blot primary antibodies

| Antibody | Concentration | Company | Catalog Number | RRID |
| --- | --- | --- | --- | --- |
| Rabbit α KCC2 | 1:1000 | Millipore | 07-432 | AB_310611 |
| Rabbit α phospho-KCC2 (S940) | 1:1000 | Aviva Systems | OAPC00188 | AB_2721198 |
| Mouse α TrkB | 1:1000 | Biosciences | 610102 | AB_397508 |
| Rabbit α phospho-TrkB (T816) | 1:500 | Millipore | ABN1381 | AB_2721199 |
| Mouse α PLCγ1 | 1:1000 | Thermo Scientific | LF-MA0050 | AB_2163544 |
| Mouse α phospho-PLC γ1 (T783) | 1:1000 | Cell Signaling Technology | 2821S | AB_330855 |
| Guinea Pig α vGLUT1 | 1:5000 | Synaptic Systems | 135 304 | AB_887878 |
| Rabbit α vGAT | 1:1000 | Thermo Scientific | PA5-27569 | AB_2545045 |
| Rabbit α phospho-CREB (S133) | 1:1000 | Millipore | 06-519 | AB_310153 |
| Mouse α β-actin | 1:10000 | LI-COR Biosciences | 926-42212 | AB_2637092 |

**Supplemental Figure 1: EEG Power vs Seizure Burden at P7.**


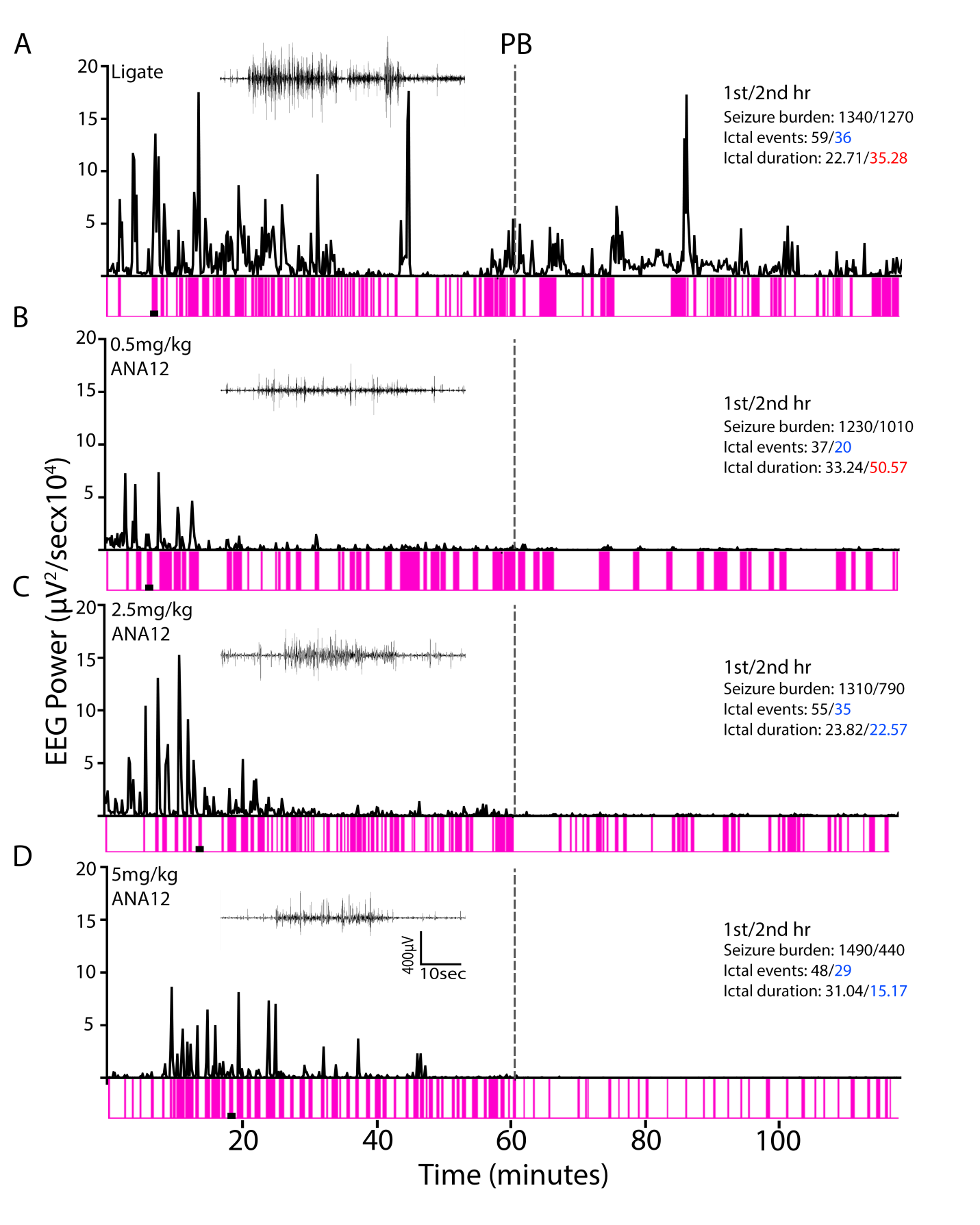


**Supplemental Figure 2:** KCC2/pKCC2 S940 downregulation and rescue 1h post-ischemia at P7


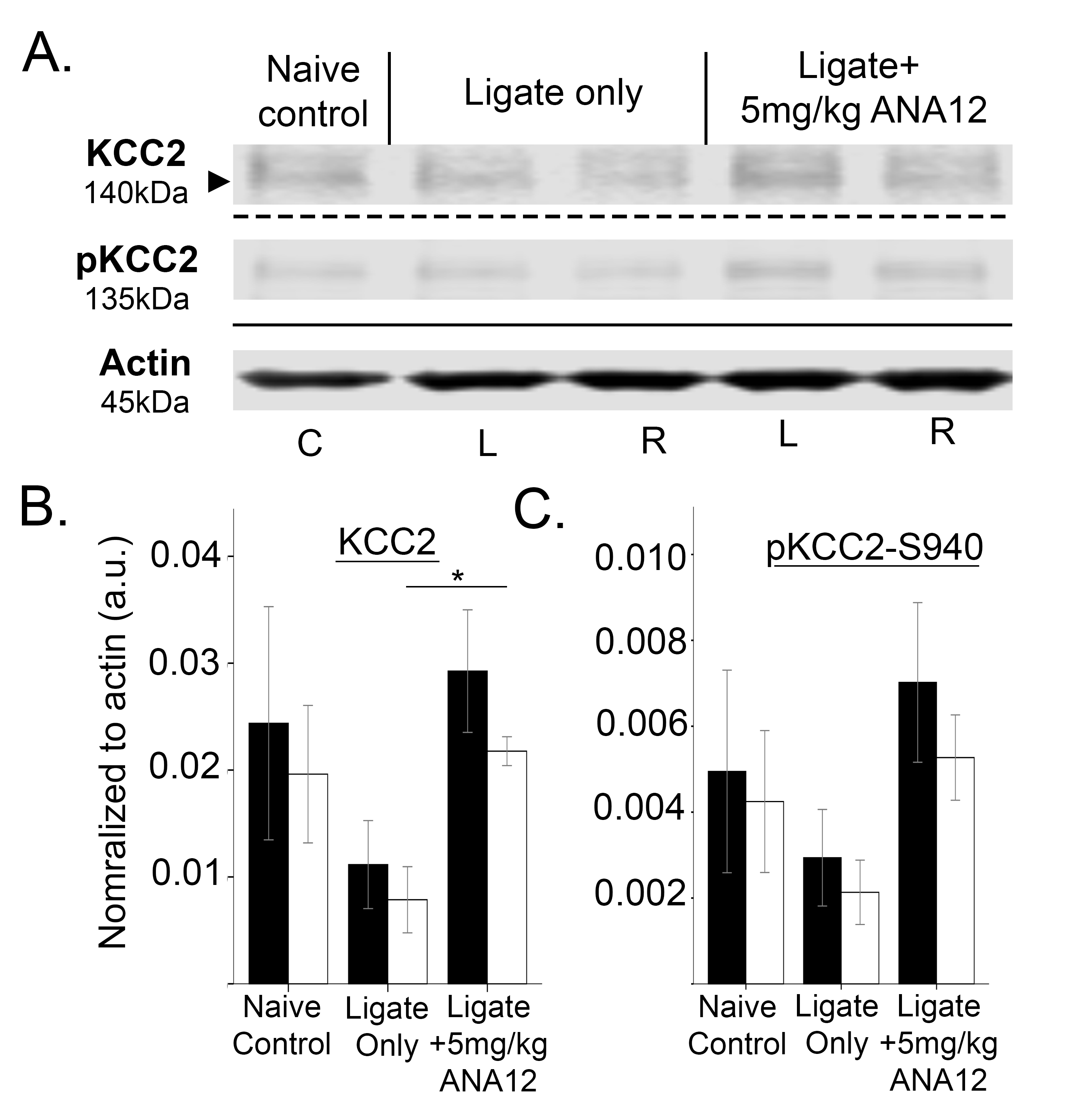


**Supplemental Figure 3:** pCREB-S133 expression 24h post-ischemia
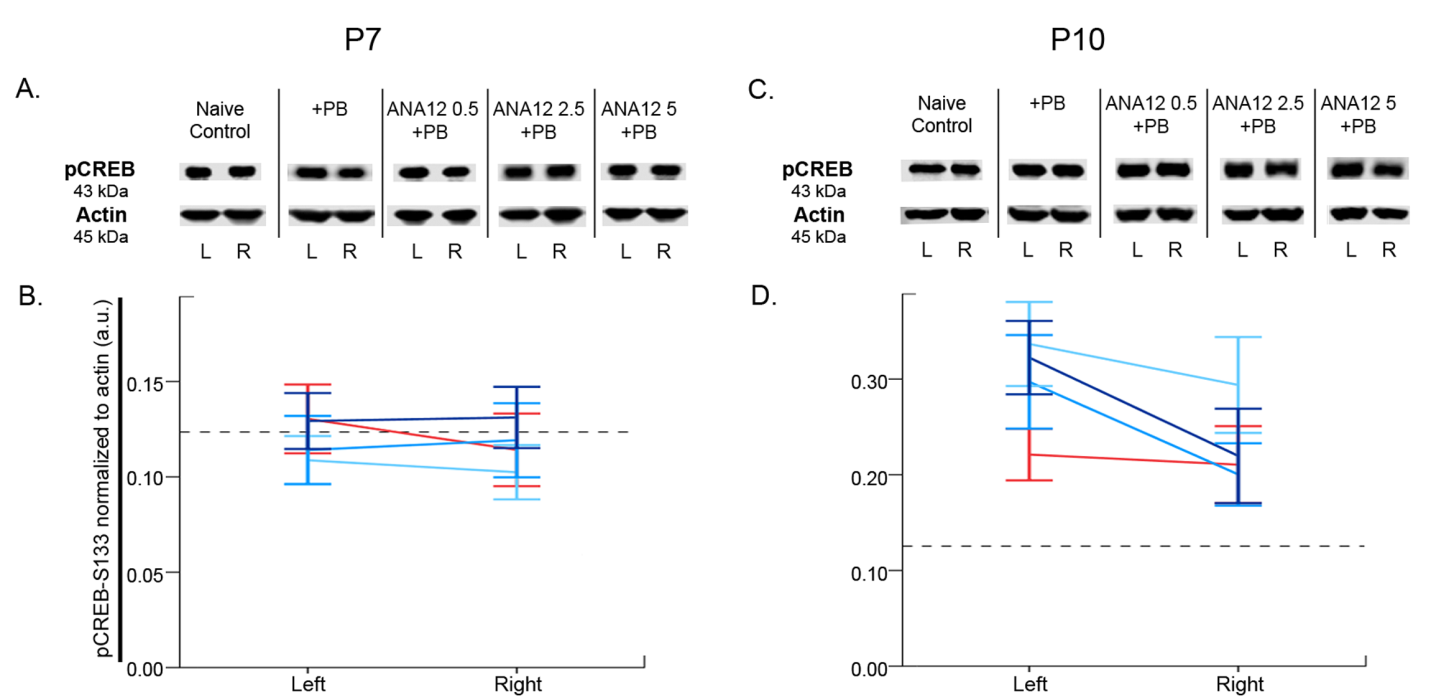


**Supplemental Figure 4:** Uncropped full image Western blots of KCC2 and pKCC2-S940 shown in Figure 4A & E.


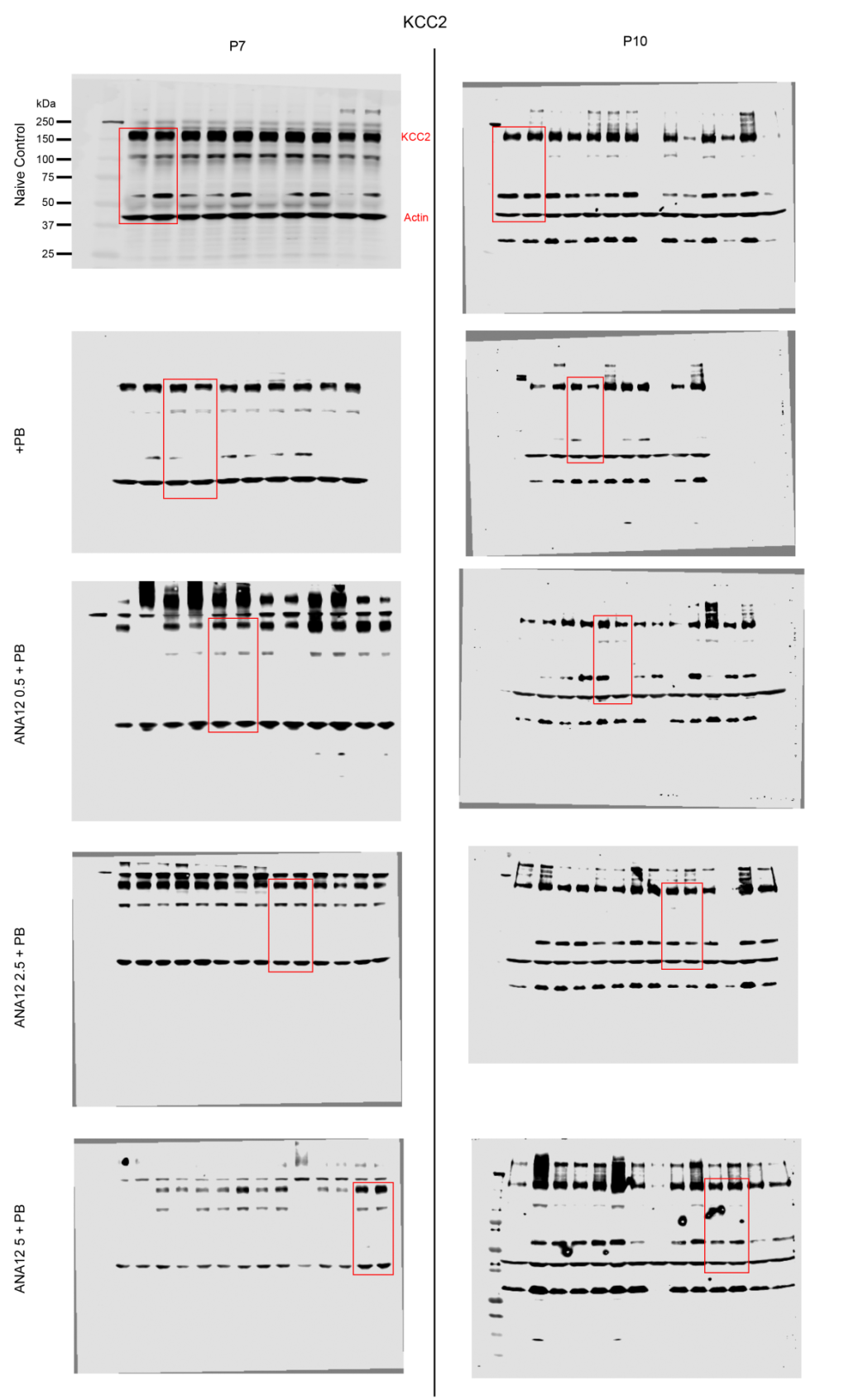


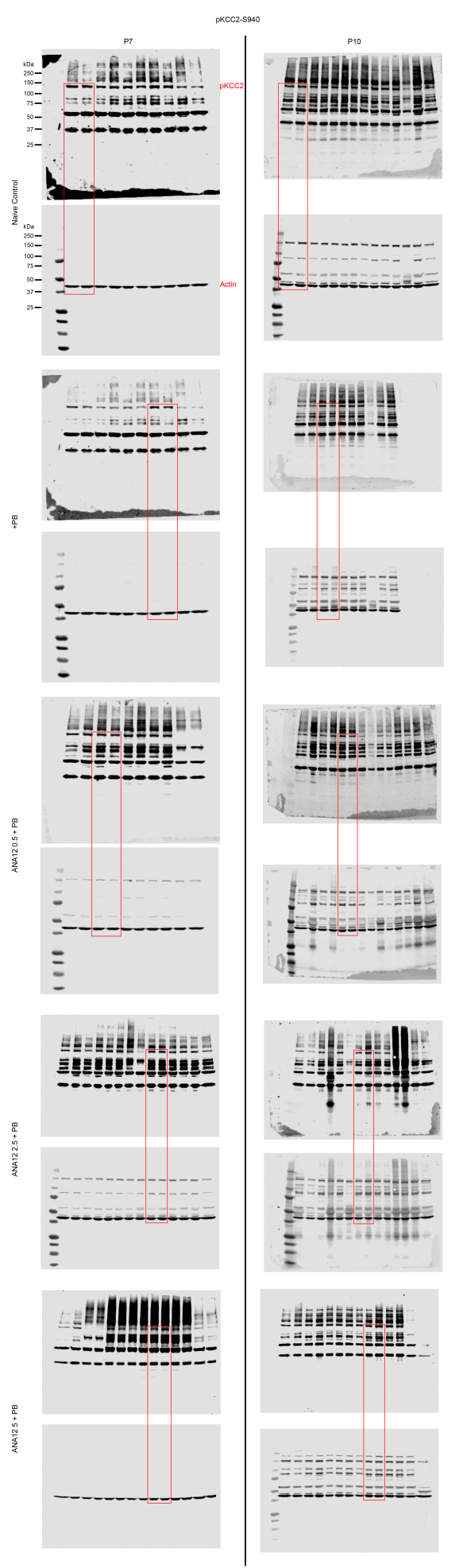


**Supplemental Figure 5:** Uncropped full image Western Blots of TrkB and pTrkB-T816 shown in Figure 5A & E.


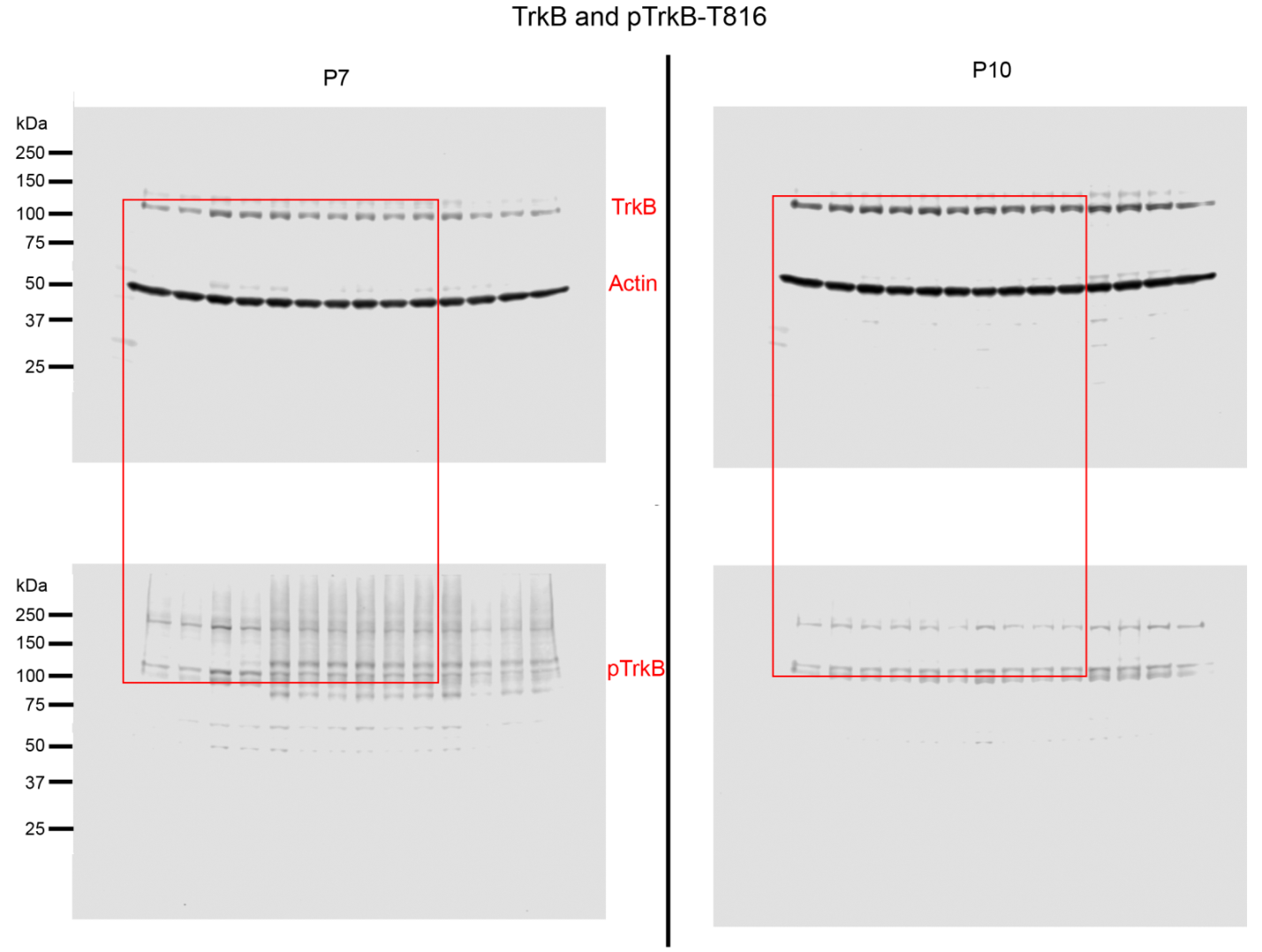


**Supplemental Figure 6:** Uncropped full image Western Blots of PLCγ and pPLCγ-T783 shown in Figure 6A & E.


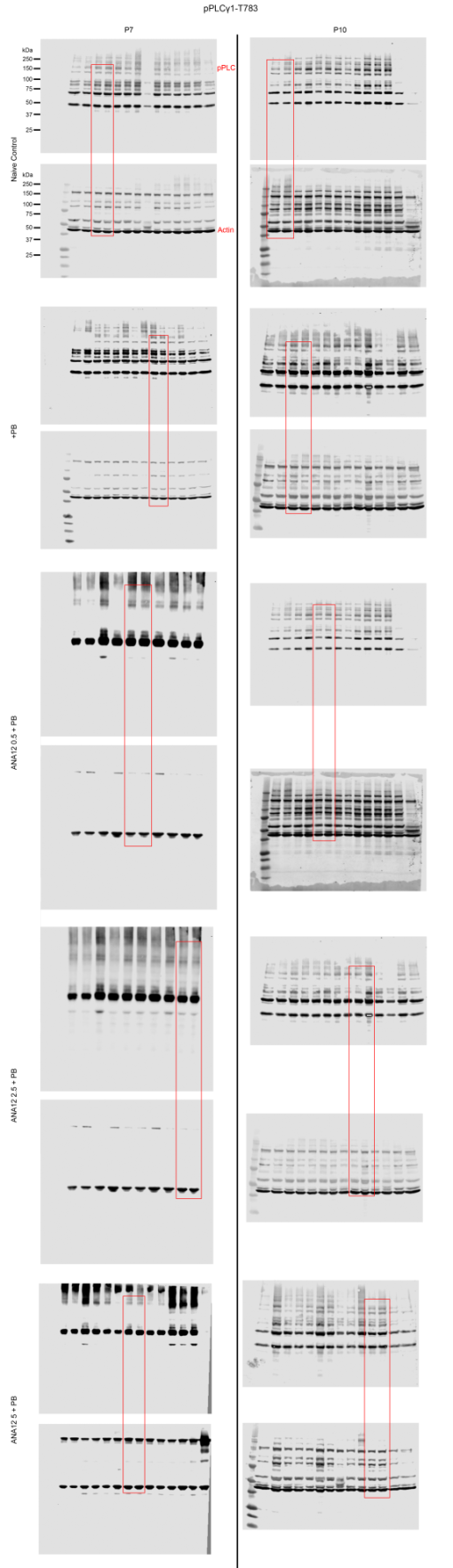


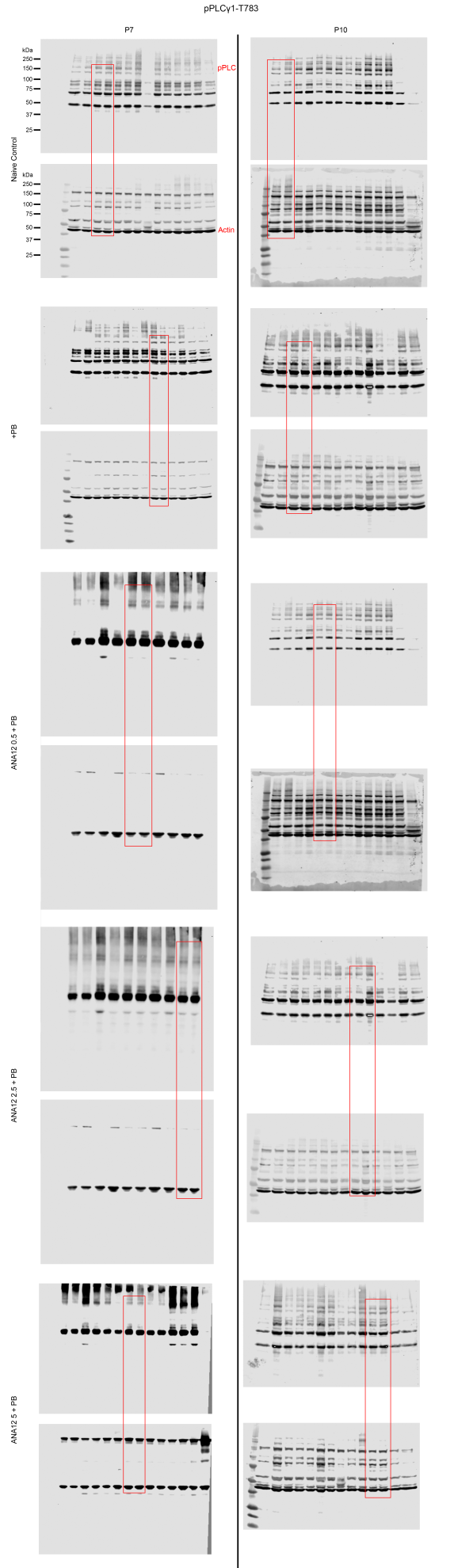


**Supplemental Figure 7:** Uncropped full image Western Blots of vGLUT1 and vGAT shown in Figure 7A & D.


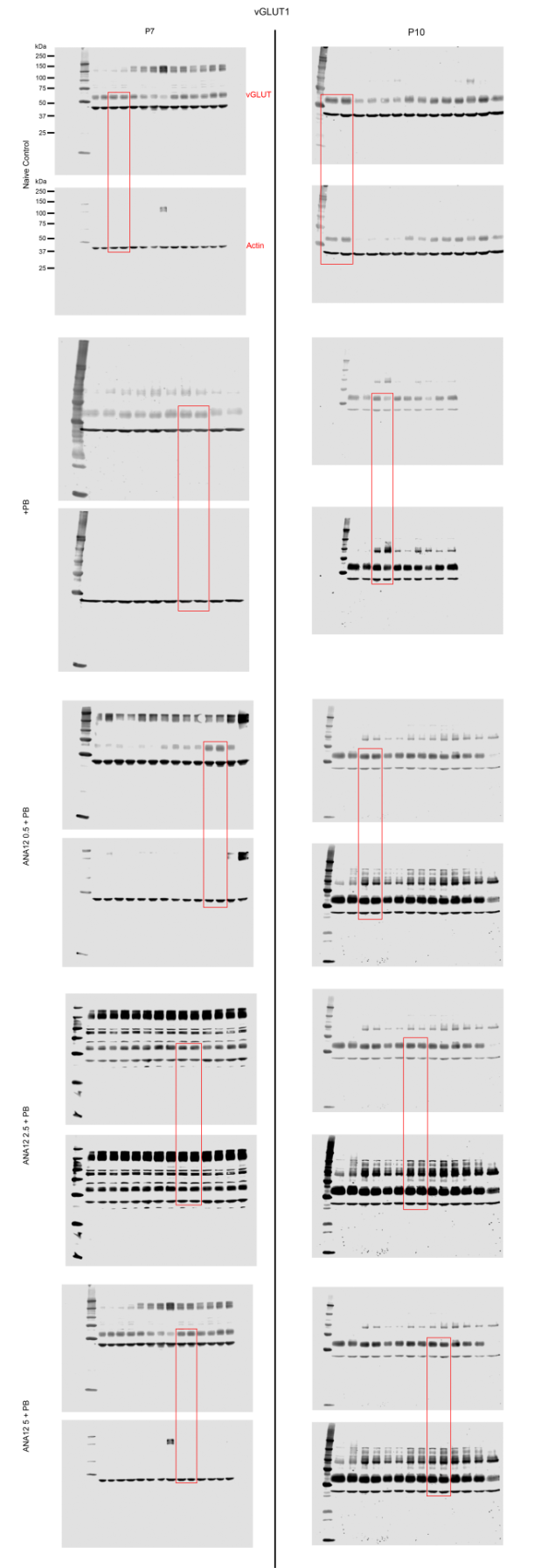


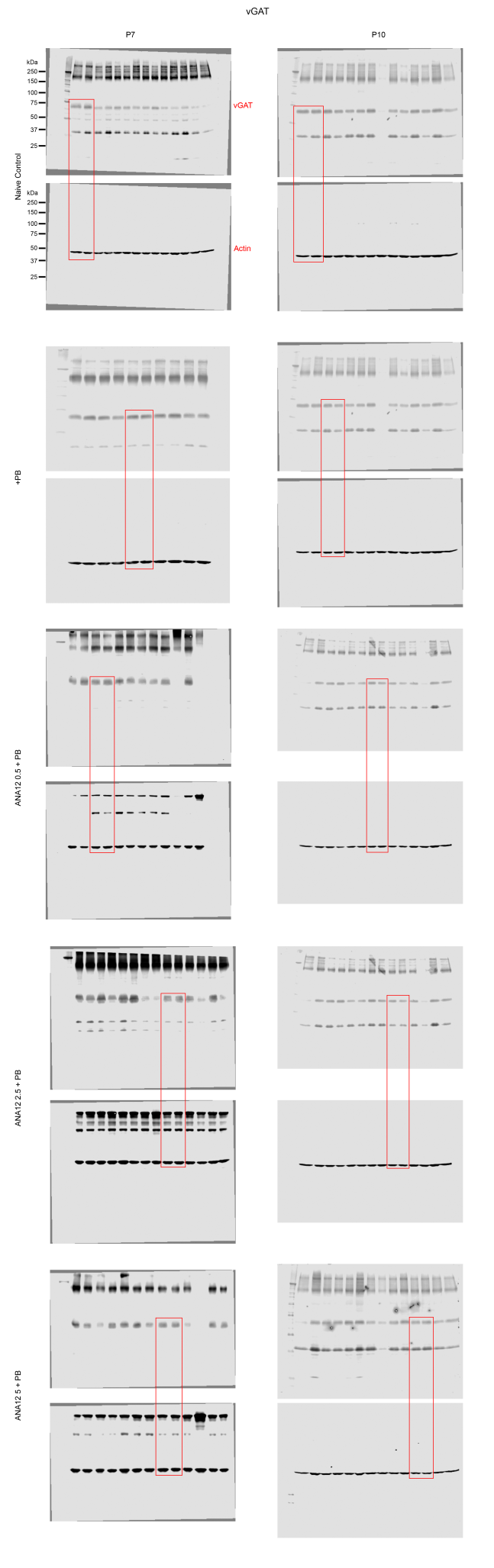


**Supplemental Figure 8:** Uncropped full image Western Blots of pCREB-S133 shown in Supplemental Figure 3A & C.


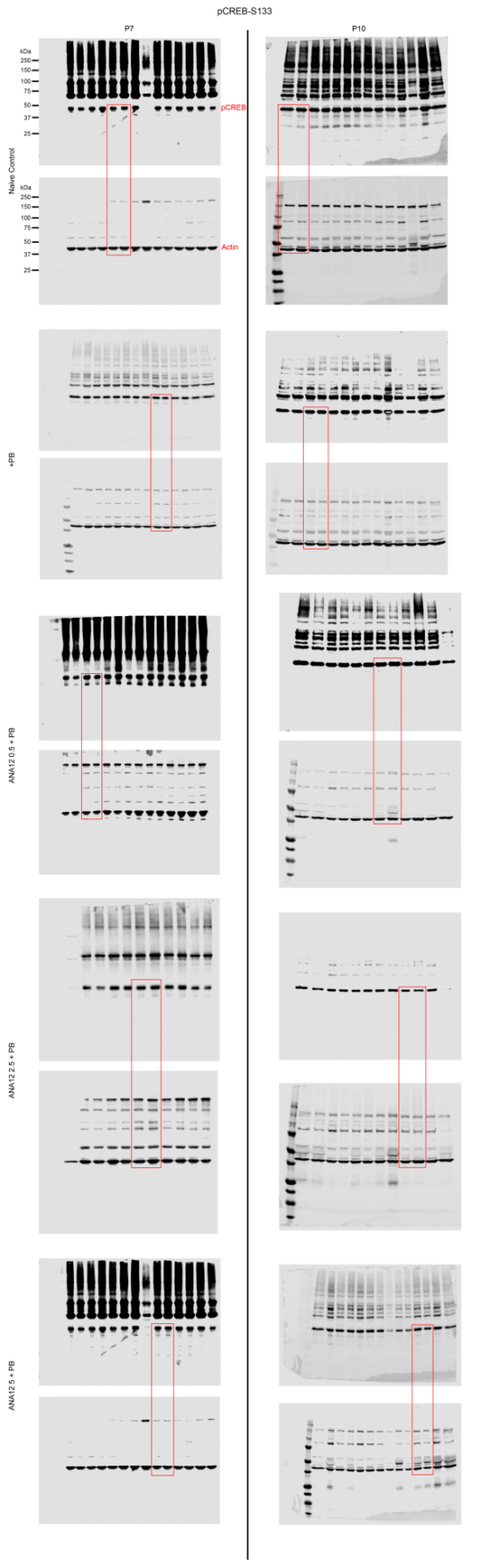


**Supplemental Figure 9:** Uncropped full image Western Blots of KCC2 and pKCC2-S940 shown in Supplemental Figure 2A.


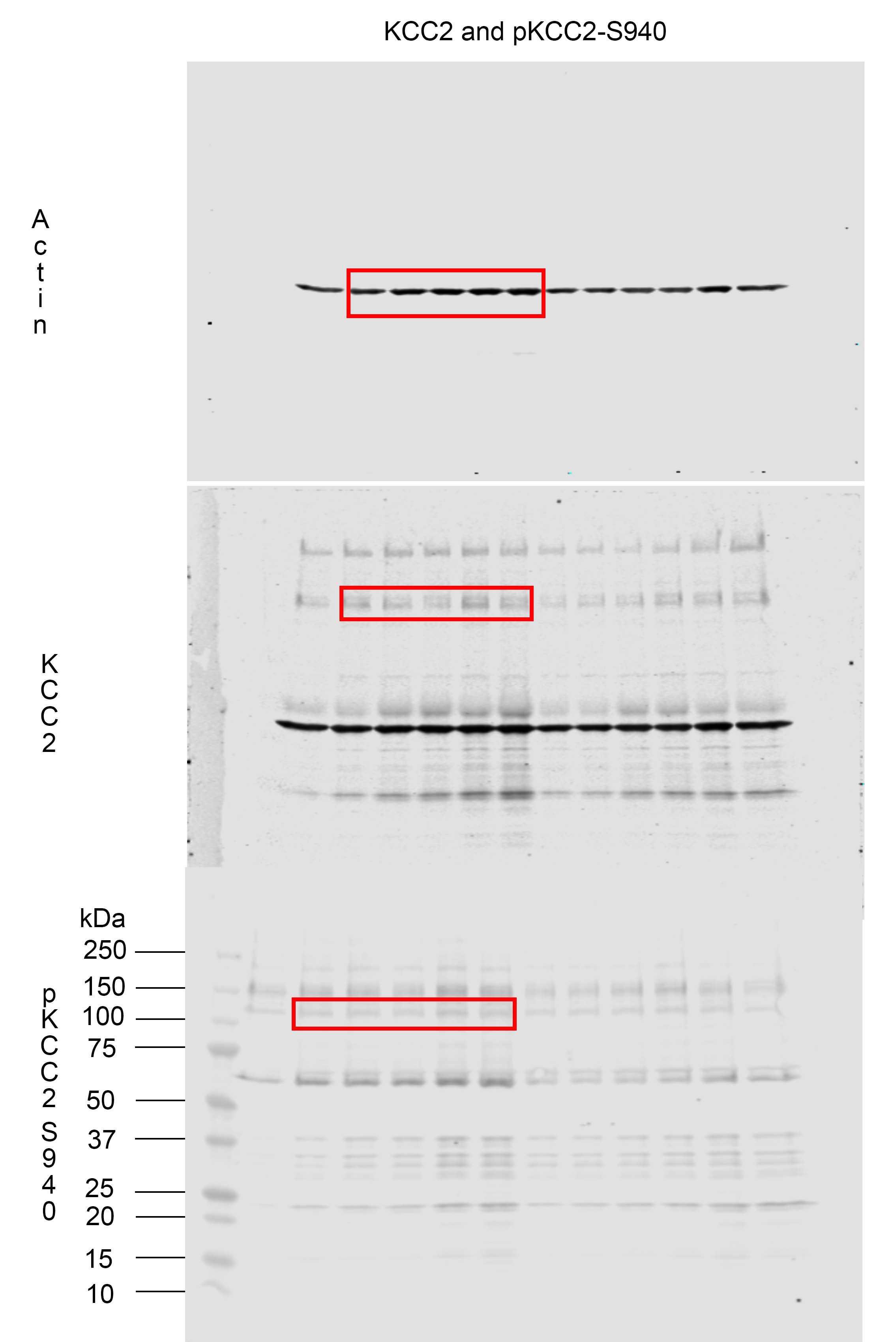


**Supplementary Video 1:** video EEG of rescue of PB-refractoriness by ANA12

(attached PPT file). P7 Video EEG of PB Only and 5 mg/kg ANA12+PB.

**Supplementary Figure 1:** EEG Power versus Seizure Burden (A-D). Representative EEG power traces for each 10-second epoch plotted with a histogram of seizures. Individual seizure traces are from the time indicated by the respective asterisk; scale in D applies to all. In the first hour, all doses of ANA12 result in a reduction of power compared to ligation alone. However, all doses of ANA12 show no effect on first hour seizure burden. Regardless of treatment, EEG power drops rapidly in the first half hour of recording. Ictal events and duration remain constant for all animals in the first hour. Put together, these results suggest that EEG power is a poor measure of anti-seizure efficacy.

**Supplementary Figure 2:** KCC2 and pKCC2-S940 expression 1h post-ischemia at P7. **A.** Representative Western blot showing KCC2 and pKCC2 expression 1h post-ischemia. **B & C.** KCC2 is lower in both hemispheres 1h post-ischemia. ANA12 rescues KCC2 degradation at 1h post-ischemia. Pups, n = 1 naïve, 1 ligate, and 2 ligate + ANA12; only male pups were used for this experiment. All samples were run in triplicate (sample size, n= 3 naïve, 3 ligate, and 6 ligate + 5 mg/kg ANA12).

**Supplementary Figure 3:** pCREB-S133 expression 24h post-ischemia. **A.** Western blots showing pCREB at P7. **B.** Quantification of blots shown in A. pCREB is not modulated by ischemia. **C.** Western blots showing pCREB expression at P10. **D.** Quantification of blots shown in C. pCREB is not modulated by ischemia at P10.
